# The Treatment of Diabetic Retinal Edema with Intravitreal Steroids: How and When

**DOI:** 10.3390/jcm13051327

**Published:** 2024-02-26

**Authors:** Maria Letizia Salvetat, Francesco Pellegrini, Leopoldo Spadea, Carlo Salati, Mutali Musa, Caterina Gagliano, Marco Zeppieri

**Affiliations:** 1 Department of Ophthalmology, Azienda Sanitaria Friuli Occidentale, 33170 Pordenone, Italy; mlsalvetat@hotmail.it (M.L.S.);; 2 Eye Clinic, Policlinico Umberto I, “Sapienza” University of Rome, 00142 Rome, Italy; 3 Department of Ophthalmology, University Hospital of Udine, 33100 Udine, Italy; 4 Department of Optometry, University of Benin, Benin City 300238, Edo State, Nigeria; 5 Faculty of Medicine and Surgery, University of Enna “Kore”, Piazza dell’Università, 94100 Enna, Italy; 6 Eye Clinic, Catania University, San Marco Hospital, Viale Carlo Azeglio Ciampi, 95121 Catania, Italy

**Keywords:** diabetic macular edema, diabetic retinopathy, vascular endothelial growth factor (VEGF), intravitreal anti-VEGF, intravitreal corticosteroids, triamcinolone acetonide, dexamethasone, fluocinolone acetonide

## Abstract

Diabetic macular edema (DME) is a common complication of diabetes mellitus and a leading cause of visual impairment worldwide. It is defined as the diabetes-related accumulation of fluid, proteins, and lipids, with retinal thickening, within the macular area. DME affects a significant proportion of individuals with diabetes, with the prevalence increasing with disease duration and severity. It is estimated that approximately 25–30% of diabetic patients will develop DME during their lifetime. Poor glycemic control, hypertension, hyperlipidemia, diabetes duration, and genetic predisposition are recognized as risk factors for the development and progression of DME. Although the exact pathophysiology is still not completely understood, it has been demonstrated that chronic hyperglycemia triggers a cascade of biochemical processes, including increased oxidative stress, inflammation, activation of vascular endothelial growth factor (VEGF), cellular dysfunction, and apoptosis, with breakdown of the blood-retinal barriers and fluid accumulation within the macular area. Early diagnosis and appropriate management of DME are crucial for improving visual outcomes. Although the control of systemic risk factors still remains the most important strategy in DME treatment, intravitreal pharmacotherapy with anti-VEGF molecules or steroids is currently considered the first-line approach in DME patients, whereas macular laser photocoagulation and pars plana vitrectomy may be useful in selected cases. Available intravitreal steroids, including triamcinolone acetonide injections and dexamethasone and fluocinolone acetonide implants, exert their therapeutic effect by reducing inflammation, inhibiting VEGF expression, stabilizing the blood-retinal barrier and thus reducing vascular permeability. They have been demonstrated to be effective in reducing macular edema and improving visual outcomes in DME patients but are associated with a high risk of intraocular pressure elevation and cataract development, so their use requires an accurate patient selection. This manuscript aims to provide a comprehensive overview of the pathology, epidemiology, risk factors, physiopathology, clinical features, treatment mechanisms of actions, treatment options, prognosis, and ongoing clinical studies related to the treatment of DME, with particular consideration of intravitreal steroids therapy.

## 1. Introduction

Diabetes mellitus (DM) is a chronic metabolic disease characterized by prolonged periods of hyperglycemia [1]. It represents a worldwide pandemic, with increasing incidence due to increased obesity and life expectancy [2]. DM represents the major cause of cardiac infarction, stroke, kidney failure, and blindness, causing high pressure on healthcare, economic, and government systems [3].

Diabetic macular edema (DME), affecting approximately 5–10% of DM patients [4], is the most important cause of vision loss in diabetes mellitus and represents the major cause of impaired vision and blindness in working-age adults in developed countries [5,6].

The pathogenesis of DME is not yet completely understood. It is thought that chronic hyperglycemia may trigger a cascade of biochemical processes, including increased oxidative stress, retinal ischemia/hypoxia, inflammation, breakdown of the blood-retinal barriers (BRBs), with accumulation of fluid inside the retina, and ultimate neurodegeneration [7,8,9,10,11,12].

Although systemic metabolic control represents the most important strategy in DME management, several different ophthalmological therapeutic approaches are now available, including macular laser photocoagulation, pharmacotherapy with intravitreal (IV) anti-vascular endothelial growth factor (VEGF) and corticosteroids (CSs), and pars plana vitrectomy (PPV) [13,14,15,16,17,18,19].

Intravitreal CSs represent a rational therapeutic option in DME, acting by reducing inflammation, stabilizing the BRBs, and reducing vascular permeability [20,21]. Triamcinolone acetonide (TA) IV injections and dexamethasone (DEX) and fluocinolone acetonide (FAc) IV sustained implants are the CSs most commonly used in DME management and have shown efficacy in reducing macular edema and improving visual outcomes in DME eyes [20,21]. However, their possible ocular side effects, in particular, the intraocular pressure (IOP) elevation and cataract development, require an extremely accurate patient selection and follow-up [20,21].

The treatment of DME with intravitreal steroids presents several clinical challenges that healthcare professionals must consider. While intravitreal steroids have shown efficacy in reducing inflammation and improving visual outcomes in patients with DME, there are concerns related to their potential side effects. One significant challenge lies in balancing the therapeutic benefits of intravitreal steroids with the associated risks, such as increased intraocular pressure (IOP) and the development of cataracts. Managing and monitoring IOP becomes crucial during the course of treatment, as elevated pressure may lead to glaucoma and further compromise vision. Additionally, the duration of efficacy and the need for repeated injections pose logistical challenges for both patients and healthcare providers. Moreover, individual patient responses to intravitreal steroids can vary, necessitating careful consideration of factors such as pre-existing conditions, the severity of DME, and the overall ocular health of the patient.

The clinical course and the prognosis of the DME are extremely variable, mainly depending on the metabolic control of the hyperglycemia, hyperlipidemia, and systemic hypertension. The response to the different therapeutic options, including the intravitreal steroids, is also highly different amongst different patients. The present manuscript aims to provide a comprehensive overview of the epidemiology, pathophysiology, diagnostic tools, treatment options, prognosis, and ongoing clinical studies related to the treatment of DME, with particular regard to IV therapy with steroids.

## 2. Methodology

The inclusion criteria limited articles to publications involving the use of intravitreal steroids in diabetic macula edema. The year of publication of articles was set to be a maximum of 5 years before writing this paper (2018). PubMed keywords were “diabetic macular edema intravitreal steroid”. Filters were further applied to sift out only articles with free full text, in the English language, and published between 2018 to 2023.

The search strategy was ((“diabetes”[All Fields] OR “diabetes mellitus”[MeSH Terms] OR (“diabetes”[All Fields] AND “mellitus”[All Fields]) OR “diabetes mellitus”[All Fields] OR “diabetes”[All Fields] OR “diabetes insipidus”[MeSH Terms] OR (“diabetes”[All Fields] AND “insipidus”[All Fields]) OR “diabetes insipidus”[All Fields] OR “diabetic”[All Fields] OR “diabetics”[All Fields] OR “diabetes”[All Fields]) AND (“macular edema”[MeSH Terms] OR (“macular”[All Fields] AND “edema”[All Fields]) OR “macular edema”[All Fields]) AND (“intravitreal”[All Fields] OR “intravitreal”[All Fields] OR “intravitreally”[All Fields] OR “intravitreous”[All Fields] OR “intravitreously”[All Fields]) AND (“steroidal”[All Fields] OR “steroidals”[All Fields] OR “steroidic”[All Fields] OR “steroids”[MeSH Terms] OR “steroids”[All Fields] OR “steroid”[All Fields])) AND ((ffrft[Filter]) AND (english[Filter]) AND (2018:2023[pdat])). A PRISMA [22] flowchart is included in Appendix A.

## 3. Diabetic Macular Edema

### 3.1. Definition and Clinical Features of the Diabetic Macular Edema (DME)

DME is defined as the DM-related accumulation of fluid, proteins, and lipids, with retinal thickening, within the macular area [5,6]

The fluid can be localized inside the retinal parenchyma (intraretinal fluid or IRF), mainly in the extracellular space of the inner nuclear, outer plexiform, and outer nuclear layers; or under the retina, between the neurosensory retina and the retinal pigment epithelium (RPE) (subretinal fluid or SRF).

DME may be focal, due to the leakage from dilated capillaries or microaneurisms, i.e., localized saccular outpouchings of the retinal capillary wall, or diffuse, i.e., related to an overall capillary hyper-permeability.

The term Clinically Significant Macula Edema (CSME), developed by the Early Treatment of Diabetic Retinopathy Study (ETDRS) [23], was defined on the basis of the slit lamp examination as follows:

- retinal thickening with 500 µm of the center of the macula;
- hard exudates within 500 µm of the center of the macula when associated with adjacent retina thickening;
- retinal thickening of 1 disc area in size, any part of which is located within 1 disc area of the center of the macula.

The term subclinical DME is used to define eyes with not clinically detectable DME but leakage or retinal macular thickening visible only with retinal imaging techniques or eyes in which the severity of DME does not reach the definition of CSME or CIDME [5,6]. DR involving the macula, named diabetic maculopathy, can be divided into an ischemic form, due to perifoveal capillary closure, or an exudative or edematous one, with the presence of DME.

DME can develop at any stage of DR; it typically occurs in mild-to-moderate non-proliferative DR [5,6]. The visual impairment related to DME has been demonstrated to be significantly linked to the edema duration and to the presence of macular ischemia, but not to the fluid amount and macular thickening [5,6]. Other DME symptoms include floaters, reduced contrast sensitivity, photophobia, changes in color vision, and scotomas, i.e., localized defects of the visual field [5,6].

### 3.2. Epidemiology and Natural History of DME

The prevalence of DR and DME is increasing worldwide due to the global epidemic of type 2 DM and the increased life expectancy, and they have been calculated to affect 35% and 7.5% of diabetic patients aged between 20 and 79 years, respectively [4].

Although there is no gender predilection, the prevalence of DME varies amongst ethnic groups, with the highest prevalence in Blacks and the lowest in Asians, and shows significant differences amongst the different countries, being the highest in North America; moreover, it appears significantly greater in subjects with type 1 DM and directly related to disease duration [4,5,6].

The Wisconsin Epidemiologic Study of Diabetic Retinopathy found that 20–25% of diabetic patients will develop a DME within 10 years after diagnosis, rising up to 30% after 20–25 years of disease duration [24].

The presence of DME also has systemic implications: it has been suggested that DM increases the risk of stroke, myocardial infarction, and cardiovascular mortality by 2- to 4-fold as compared to healthy subjects and that the presence of DME increases the risk of arterial thromboembolic events (ATEs) by 2-fold as compared to diabetic patients without DME [25]. Thirty percent (30%) of eyes with subclinical DME have been demonstrated to progress to CSME over a median follow-up of 14 months [5,6].

The ETDRS study was a multicenter RCT that included 3711 DM patients and was designed to assess whether argon laser photocoagulation can reduce the risk of visual loss in patients with DR. The study showed that, over 3 years of follow-up, untreated DME eyes with BCVA ≤ 5/10 showed a VA gain of ≥6 letters in 20–25% of cases and a VA loss of ≥15 letters in 25% of cases. VA loss rate was inversely related to the baseline VA and directly related to the DR severity [23].

The Diabetic Retinopathy Clinical Research. Network (DRCR.net) Protocol V, a large randomized controlled trial (RCT) that enrolled eyes with CIDME and preserved BCVA (>20/32), found no significant differences in vision loss between prompt anti-VEGF, laser therapy, or observation at 2-year follow-up, with 30% of eyes showing a spontaneous resolution of CIDME by 2 years [26].

DME-related visual dysfunction may be reversible in the short term if adequately treated, even with metabolic control alone, but long-standing DME may induce irreversible retinal damage, with neuronal and RPE alterations and sub-retinal fibrosis, resulting in permanent visual loss [27]. However, the presence of an associated ischemic diabetic maculopathy represents a negative predictive factor for spontaneous visual recovery and response to therapies [5,6].

### 3.3. Diagnosis

DME diagnosis is made by finding an ME due to DM on the fundus examination. Diagnostic methods useful for DME diagnosis include the following:

**a**  **Stereoscopic slit-lamp examination of the fundus using a Volk fundus lens**: This is the most used method of diagnosing DME worldwide.
**b**  **Color and stereo fundus photographs:** These may be used to document clinical findings to stage DR and DME and identify their progression by comparing images longitudinally.
**c**  **Fluorescein angiography (FA):** Before the introduction of the OCT, FA has been the only method to assess and classify DME for several years [28]. FA retains fundamental importance in DME evaluation because it is the only imaging method able to detect vascular leakage and identify non-perfused areas and neovascularization in the retinal periphery [28], whose identification is mandatory to classify the DR severity and guide the treatment [4]. Non-perfused peripheral areas may indeed release inflammatory mediators that can sustain and explain a persistent DME, and the laser ablation of these ischemic areas may improve the central retinal morphology and function [29].

As stated by the European Society of Retina Specialists (EURETINA) guidelines published in 2017, FA remains the gold standard in assessing DME prior to any treatment to exactly stage DR and DME and should be repeated in the event of non-responsiveness to therapy [14].

**d**  **Optical Coherence Tomography (OCT):** This non-invasive, highly reproducible imaging method represents the new gold standard in DME diagnosis and follow-up [28,30], and the current guidelines suggest that OCT should be routinely used in the clinical evaluation of all DR patients [14].

DME is diagnosed on OCT as intra-retinal and/or sub-retinal hypo-reflective spaces. A central retinal thickness (CRT) > 250 µm in combination with retinal thickening is generally considered to be macular edema [30]. A recent systematic review and meta-analysis reported that the overall worldwide prevalence of DME diagnosed using OCT is 5.5% [31]. In about 44% of cases, DME is visible on FA or on OCT but not on both [28].

Moreover, previous studies have identified OCT morphologic biomarkers that have shown prognostic value for visual function recovery and could guide the choice of the different therapeutic options [32]. OCT variables, which are highly inversely related to DME eyes’ visual function and represent prognostic indicators of poor treatment response, include the following: disorganization of the inner retinal layers (DRIL), alterations of the inner and outer photoreceptor segment lines, alteration of the internal and external limiting membranes, presence of exudates, and hyper-reflective foci [32]. The parameters “CRT” and “central subfoveal thickness (CST)” are poorly associated with baseline BCVA or with VA change after therapy, and the correlation decreases with increasing DME duration and the presence of ischemia [32]. Considering that VA measurements can have low reproducibility, they remain the gold standard in evaluating the efficacy of the different treatment approaches [14,16,17]

**e**  **OCT angiography (OCT-A):** This provides high-reproducible and high-resolution images of the retinal vasculature, segmented in the different retinal layers that cannot be individually visualized on FA, without intravascular dye injection. OCT-A is able to show modification of the foveal avascular zone dimension, identify areas of capillary non-perfusion, and image the deep capillary plexus, which is not visible on FA, allowing a more accurate DR classification and providing useful data of the response to the different therapies [33].

The differential diagnosis of DME includes all other causes of ME [34], including the following: intraocular surgery, venous occlusive diseases, posterior segment inflammatory diseases, intraocular tumors, use of local or systemic drugs, etc. An accurate anamnesis and the presence of other clinical features of DR are of fundamental importance for a correct diagnosis.

### 3.4. Pathophysiology of DME as Rationale for Different Therapeutic Strategies

The pathophysiology of DME is highly complex and still not completely understood. The metabolic and oxidative stress caused by chronic hyperglycemia in vitreous, retinal, and choroidal cells is thought to induce four main mechanisms leading to DME development and progression [7,8,9,10,11,12]:

**a**  **Retinal ischemia/hypoxia**: This is considered to be the *primum movens* in DME pathogenesis. It is related to the retinal capillary and arterioles closure due to the pathological adhesion of altered leucocytes to a damaged vascular endothelium. This phenomenon, called leukostasis, seems to play an early and fundamental role in diabetic micro-vasculopathy development [8]. The most important consequence of retinal ischemia/hypoxia seems to be the up-regulation of many pro-inflammatory molecules [7], including the VEGF, which is considered to be the single most important mediator in DME pathogenesis [10,12]. VEGF causes inner BRB breakdown with consequent vascular hyper-permeability; it acts as a potent pro-inflammatory agent and may induce retinal neovascularization, it being a potent mitogen of the vascular endothelial cells [35].
**b**  **Inner and outer blood-retinal barriers (BRBs) breakdown**: In DME eyes, the long-standing hyperglycemia causes increased apoptosis of pericytes, vascular endothelial cells, EPR, and Mueller glial cells, along with diffuse damage of the intercellular tight-junction. The consequences are the breakdown of both inner and outer BRBs and the dysfunction of the active cellular transport of fluids out from the retina carried out by Mueller glial cells and RPE. The inner BRB breakdown seems to play a major role in DME pathogenesis [7,8].
**c**  **Low-grade of chronic retinal inflammation:** Macular edema is considered to be the most important clinical manifestation of retinal inflammation, and several studies have shown that inflammatory mechanisms play a fundamental role in DME development and progression [7,8,9,10,11,12].

Retinal cells, damaged by the long-standing hyperglycemia and by the subsequent retinal ischemia/hypoxia, have been demonstrated to release various pro-flogistic and pro-angiogenic mediators and activate many inflammatory and immune cells, such as macrophages, leucocytes, and retinal microglia. The inflammatory cascade increases oxidative stress and cellular damage, creating a vicious circle (Table 1).

Aqueous, vitreous, and retinal concentrations of the inflammatory mediators involved in DME pathogenesis have been demonstrated to correlate with DME severity, amount of leakage on FA, and CRT measured by OCT [32].

These data suggest that, although VEGF seems to be the central player in DME pathogenesis [10,12], several other VEGF-independent retinal inflammatory pathways are up-regulated and likely exert an important role [7,8,9,10,11,12].

**d**  **Increased retinal neurons degeneration and apoptosis:** This has been demonstrated to occur early in DME eyes and to be independent of the microvascular alterations [36]. Several pieces of evidence suggest that retinal neurodegeneration may be a consequence of different factors, including chronic low-grade retinal inflammation, micro-environment alterations, glutamate accumulation, and oxidative stress. The damage and apoptosis of the retinal neurons cause a reduced retinal function and, if left untreated, will result in permanent visual loss [36].

### 3.5. Therapeutic Approaches in the Management of DME

The treatment of DME represents a clinical challenge. Although the control of systemic risk factors represents the most important strategy in DME management, several other specific therapeutic options are now available [13,14,15,16,17,18,19]: macular laser photocoagulation; pars plana vitrectomy; and IV pharmacotherapy with anti-VEGF agents or corticosteroids, which is currently considered as the first-line approach in DME management [37,38].

The therapy of DME should be personalized to each patient based on the evaluation of several factors, including DME localization (CIDME or non-CIDME), severity (CIDME with or without visual loss), DME type and duration, FA, OCT, and OCT-A features, responsiveness to previous treatments, associated general and ophthalmological comorbidities, and patient compliance.

Considering that the final BCVA in DME-treated eyes appears directly related to the baseline BCVA [39], early diagnosis and timely treatment have been shown to be crucial for correct DME management.

The current guidelines for the treatment of DME can be summarized as follows [13,14,15,16,17,18,19]:

- **Non-center involving DME (non-CIDME):** As suggested by the ETDRS trial [23], the management approaches may include observation until the fluid involves the fovea or laser therapy in the presence of a considerable amount of edema/exudates located far from the fovea, i.e., >500 µm from the foveal avascular zone.
- **Center-involving macular edema (CIDME) with preserved VA (VA > 20/32):** CIDME eyes with preserved vision are treated on the basis of the results of the DRCR.net Protocol V, an RCT that randomized eyes with CIDME and VA ≥ 20/25 to receive either prompt anti-VEGF, prompt macular laser or observation, and did not find differences amongst the three groups [26]. In these cases, observation, focal macular laser, IV injections of anti-VEGF or CSs, or a combination of these options are suggested.
- **Center-involving macular edema (CIDME) with visual loss (VA ≤ 20/32):** CIDME eyes with visual loss were eligible for several RCTs that have demonstrated that the IV injections of anti-VEGF or CSs are significantly more effective than observation or laser for DME treatment, leading to the approval of the currently used IV pharmaceutic agents [40,41,42,43].

The current guidelines consider IV pharmacotherapies with anti-VEGF and/or CSs as the gold standard of the treatment of CIDME with visual loss [13,14,15,16,17,18,19].

### 3.6. The Role of Metabolic Control, Laser Photocoagulation, Pars Plana Vitrectomy, and Intravitreal Anti-VEGF in DME Management

**a**  **Metabolic control:** The most important strategy for the prevention and treatment of DR and DME remains the control of the systemic risk factors favoring DME development and progression, including hyperglycemia, hyperlipidemia, systemic hypertension, anemia, and other hematological disorders, renal failure, sleep apnea, and carotid artery stenosis [4].

The Diabetes Control and Complications Trial [44] and the United Kingdom Prospective Diabetes Study [45] have demonstrated that an accurate control of glycemic levels (HbA1c ≤ 7), blood pressure, and serum lipids is able to reduce the cumulative incidence of DME and the need of laser treatment in diabetic patients.

The optimization of the systemic factors control may require a multidisciplinary team involving ophthalmologists, endocrinologists, and internal medicine specialists.

**b**  **Laser photocoagulation:** Focal photocoagulation of the leaking microaneurisms or grid laser photocoagulation of areas with diffuse leakage of the capillary bed has represented the gold standard in DME treatment from the mid-1980s for approximately 30 years [23] until the introduction of the intravitreal anti-VEGF therapy [41,43]. The mechanism of action of laser photocoagulation in the treatment of DME has not been fully elucidated. Focal photocoagulation laser likely works by destroying the sources of fluid leakage such as microaneurisms, but it probably may also improve the cytokine release from the RPE or Mueller cells and the RPE active pump of fluid toward the choroid. The grid laser increases the oxygenation of the inner retina by both decreasing the number of photoreceptors that are oxygen-consuming and favoring the diffusion of oxygen from the choroid to the inner retina.

The efficacy of the focal/grid laser for DME treatment has been validated by the ETDRS study in the 1980s [23] and by the DRCR.net Protocol B [46], which demonstrated that CSME eyes treated with focal/grid laser photocoagulation had reduced risk of moderate visual loss in 50% of cases as compared with the disease natural history.

Laser photocoagulation for CIDME management had several limitations: it did not specifically address the underlying causes of DME; it was associated with limited or no visual function improvements; and it showed that potential side effects may cause irreversible retina damage, including central or paracentral scotomas, subretinal fibrosis, and secondary choroidal neovascularization [23,46].

Aiming to decrease the thermal destructive effect on RPE and photoreceptors related to the conventional lasers, subthreshold micropulse grid laser (yellow or infrared) has been recently proposed as an alternative or adjuvant for DME treatment [47,48]. This treatment option, which likely acts by improving the RPE function and normalizing the retinal inflammatory pathways, has shown a high safety profile and efficacy comparable to conventional lasers. No standardized treatment parameters are available, the response to treatment is very slow, and several treatments are necessary to achieve DME elimination [14,15].

Conventional and subthreshold micropulse lasers are actually considered as a second-line or adjunctive treatment in selected DME eyes and are mostly used in cases of non-CIDME [13,14,15,16,17,18,19].

Laser therapy, however, has a longer duration than IV pharmacotherapies. It can be particularly useful in developing countries, where access to IV drugs is limited, or when IV pharmacotherapies are contraindicated, such as during pregnancy or breastfeeding [13,14,15,16,17,18,19].

**c**  **Pars plana vitrectomy (PPV):** Although still controversial, the vitreomacular traction seems to be a relevant factor in the development and maintenance of DME [10]. Animal and clinical studies have suggested that PPV may work by reducing vitreoretinal tractions, increasing vitreal and retinal oxygenation, and cleaning the vitreous from inflammatory mediators including VEGF, representing, therefore, a rational approach in DME eyes [49,50].

PPV can be considered in cases of DME associated with vitreomacular traction, epiretinal membranes, or the presence of proliferative diabetic retinopathy (PDR) with macular traction. Although previous authors have demonstrated the efficacy of the PPV in terms of VA gain in DME eyes with vitreomacular traction, the peeling of epiretinal or internal limiting membranes has provided structural improvements in the absence of significant VA gain [49,50]. Therefore, the advantages of PPV in DME patients without vitreomacular traction remain uncertain and should be considered only in cases when the response to IV anti-VEGF or steroids is unsatisfactory [13,14,15,16,17,18,19].

**d**  **Intravitreal anti-VEGF:** The IV injections of anti-VEGF are currently considered as the first-line treatment of CIDME [13,14,15,16,17,18,19].

The introduction of the anti-VEGF in the 2000s has indeed changed the treatment paradigm because, as compared to laser photocoagulation, the intravitreal anti-VEGF therapy was not only able to reduce the risk of vision loss but also to increase visual acuity [41,43], an outcome uncommonly seen after laser therapy [23,46].

VEGF has been demonstrated to be the most important single factor responsible for DME pathogenesis, and its intraocular concentration has been found to correlate to DME severity [7,10,12], which provides a rationale for the use of the anti-VEGF in DME management. Anti-VEGF is indeed able to antagonize the VEGF, reducing vasodilation, vascular hyper-permeability, retinal edema, and neovascularization induced by the VEGF [51].

Several anti-VEGF molecules with different weights, structures, binding affinities, and targeted VEGF isoforms, are currently available for DME treatment, including the following:

- Bevacizumab (Avastin, Genentech Inc., San Francisco, CA, USA) [52]: This is a full-length recombinant humanized monoclonal antibody binding the VEGF-A, approved for the treatment of several tumors, including metastatic colon, rectum, or breast cancer, that is used off-label in ophthalmology.
- Ranibizumab (Lucentis, Novartis, Basel, Switzerland) [41,53]: This is a recombinant humanized monoclonal antibody fragment that binds VEGF-A. It was the first anti-VEGF approved in 2012 for the treatment of DME.
- Aflibercept (Eylea, Bayer, Leverkusen, Germany) [43,54]: This is a fusion protein that binds and inhibits all VEGF-A, VEGF-B, and PIGF isoforms. Aflibercept comes in 2 mg- and 8 mg-formulations.
- Brolucizumab (Beovue, Novartis): This is a new VEGF-A antagonist with a smaller molecular weight and a longer duration of action [55].
- Faricimab (Vabysmo, Genentech/Roche): This is a new monoclonal antibody targeting both VEGF and Angiopoietin-2 [56].

Multicenter RCTs have demonstrated that IV anti-VEGF therapy is superior over no treatment, placebo treatment, or laser photocoagulation in the management of CIDME, with visual loss, with an acceptable safety profile [41,43,53,54]. Anti-VEGF agents have been demonstrated to provide BCVA improvements of ≥ 3 Snellen lines in approximately 30–40% of cases [57], usually developing with the first 3–6 monthly injections [58].

IV anti-VEGF drugs have several drawbacks:

- In total, 40–65% of patients show persistent DME despite regular IV injections of anti-VEGF [53,59,60,61], suggesting that other factors beyond VEGF might play a role in the pathogenesis of DME.
- Anti-VEGF IV injections are characterized by short durations of action, with high rates of DME recurrence and pronounced CRT fluctuations [41,43,51,53,54,59,60,61], that have been demonstrated to be risk factors for irreversible retinal damage and visual loss [62,63]. Moreover, the need for frequent repeated IV injections leads to patients’ fatigue and lack of adherence; high costs for the public health service; and increased risk of side effects related to the intravitreal injections, such as infectious endophthalmitis, intraocular inflammation, iatrogen cataract, vitreal, and retinal hemorrhages [64].
- Anti-VEGF therapy has been associated with several systemic side effects, including deterioration of systemic hypertension, kidney disease, gastrointestinal perforation, stroke, and myocardial infarction [57,65,66].
- Anti-VEGF agents have been linked with the risk of persistent IOP rise requiring IOP-lowering treatment (5–10% of cases); with cataract development (0–15% of cases); and with the decrease of retinal ganglion cells layer thickness, which seems directly related with the number of IV anti-VEGF injections [66].

## 4. Corticosteroids for the Management of DME

### 4.1. The Corticosteroids

**a**  **Definition**

Corticosteroids (CSs) are a group of hormones produced by the adrenal cortex; they are classified into glucocorticoids, including cortisol and cortisone, that regulate the metabolism of carbohydrates, proteins, and lipids and mineralocorticoids, such as aldosterone, that control salt and water balance in the body [67,68].

**b**  **History of the CSs’ pharmacological use**

The idea to use glucocorticoids to treat inflammatory diseases dates back to 1948 and was related to the observation that rheumatoid arthritis had a tendency to improve during pregnancy and in patients affected by jaundice, with both conditions being characterized by high glucocorticoid levels [67,68]. After that, CSs were used to treat several different inflammatory diseases, and ophthalmologists introduced their use to treat uveitis in the early 1950s [67,68]. During the last 70 years, several new steroids have been synthesized and released for therapeutic use. The first treatment of DME with CSs was published in 2001 [69].

**c**  **Biological effects**

CSs have extremely complex biological effects that involve the regulation of multiple genes. Both endogenous and synthetic steroids bind specific glucocorticoid receptors and regulate the expression of approximately 10–20% of the human genome in almost all cell types, resulting in glucose metabolism, growth, development, survival, and inflammation control [67,68]. Different steroid molecules differ in molecular weight and structure, receptor binding affinity, and gene modulation pattern profile. Their biological properties and side effects vary in different cells and different subjects depending on many variables that may explain the resistance or hypersensitivity to steroids, including receptor expression, receptor polymorphisms, sex, and disease variables, such as the glycemic status, and therapy duration [67,68].

**d**  **Therapeutic effects**

Corticosteroids are commonly prescribed with a variety of indications due to their wide range of effects on the human body [67,68]. Because of their anti-inflammatory and immune-suppressive effects, CSs are used to treat many inflammatory, allergic, and autoimmune diseases, including asthma, allergic rhinitis, hay fever, urticaria, atopic eczema, chronic obstructive pulmonary disease, rheumatoid arthritis, lupus, Crohn’s disease, ulcerative colitis, giant cell arteritis, polymyalgia rheumatic, multiple sclerosis, inflamed joints, muscles and tendons, non-infective uveitis, etc. [67,68].

**e**  **Side effects**

Steroids have many side effects targeting different tissues and organs, including hypertension, dyslipidemia, glucose intolerance and diabetes mellitus, obesity, hirsutism, gastrointestinal irritation, peptic ulcer, osteoporosis, delayed wound healing, increased risk of infections, virus reactivation, fluid retention, growth retardation, hypothalamic-pituitary axis suppression, mood disturbance, depression, insomnia, psychosis, etc. [67,68]. The CSs’ side effects on the eye include mainly ocular hypertension (OHT), glaucoma and cataract development, and, less frequently, central serous chorioretinopathy (CSCR) and infections reactivation [70,71,72,73,74,75].

- Steroid-induced OHT and steroid-induced glaucoma (SIG) are the most frequent and dangerous side effects of the systemic and, most frequently, local use of CSs [74]. Subjects who respond to treatment with glucocorticoids with an IOP rise are referred to as “steroid-responders”, whose definition is not univocal and may include the following cases: IOP increase of >5 mmHg or >10 mmHg from baseline or IOP > 21 o 24 mmHg [74]. SIG can be considered to be a dangerous form of secondary open-angle glaucoma because it is frequently diagnosed late and is characterized by IOP levels that can be particularly high and lead to significant optic disc and perimetric damages within a short time [74].

The pathogenesis of the steroid-induced OHT and SIG is still unclear. It has been demonstrated that steroids regulate the expression of several genes at the level of the trabecular meshwork and can cause an increased aqueous humor outflow resistance by both increasing deposition of extracellular matrix proteins as well as inducing trabecular meshwork cell dysfunction [74].

The prevalence of the steroid-induced OHT and SIG is variable. Considering a normal population, approximately 61–63% can be classified as non-responders, showing an IOP rise of <5 mmHg; 33% are low-moderate responders, with an IOP elevation ranging from 6 to 15 mmHg; and 4–6% are high responders, with an IOP increase of >15 mmHg [76]. On the other hand, amongst glaucomatous patients, 46–92% show a significant IOP rise after topical steroid administration [76].

Risk factors for the development of steroid-induced OHT and SIG are as follows: individual susceptibility, likely related to different isoforms of the glucocorticoids receptors; older and younger age, especially children younger than 6 years; glaucomatous patients and first-degree relatives of glaucomatous patients; connective tissue diseases; and high myopia, DM type I [74].

Steroid-induced OHT is usually reversible by the interruption of CS therapy, with IOP usually returning to normal levels in 2–4 weeks after discontinuing the steroids [74].

- Steroid-induced cataract: Prolonged use of high doses of CSs, especially if systemically administered, is a significant risk factor for the development of bilateral posterior subcapsular cataracts, with a higher incidence in children and susceptible subjects [73]. CSs are the fourth leading risk factor for cataract development, following diabetes, myopia, and glaucoma, and it has been calculated that approximately 4.7% of all cataracts are steroid-induced [73].

The mechanisms underlying lens opacification, also when associated with CS-therapy, are still unknown. It is supposed that the steroid-induced reduction of the VEGF and other growth factors in the aqueous humor may prevent the normal differentiation of the lens epithelial cells into fiber cells. The undifferentiated lens epithelial cells migrate along the capsule until reaching the posterior pole, where they form an irregular aggregate of cells that scatter light [73]:

- Because of their immunosuppressive effect, steroids may favor opportunistic bacterial, viral, and fungal ocular infections that are most often associated with the topical use of CSs [70,75].
- Central serous chorioretinopathy (CSCR) development or recurrence: CSCR is an idiopathic retinal disease characterized by leakage of fluid through the RPE into the subretinal space, with serous detachments of the neurosensory retina and RPE, leading to central vision loss and metamorphopsia. Although still debated, the association between CRSC and CSs has been widely reported [71,72]. The proposed pathogenic mechanisms are a steroid-induced RPE active fluid pump impairment and an increase in the choroidal vessel permeability [71,72].

**f**  **The rationale of the use of steroids in the pharmacological approach to DME**

CSs have gained great interest in DME management over the last years, and the rationale for their use is clear evidence that inflammation plays a fundamental role in DME pathogenesis and that several inflammatory pathways beyond VEGF are involved in this process [7,10,12].

CSs represent an alternative therapeutic strategy in DME because of their multiple anti-inflammatory and anti-angiogenic effects [20,21] (Table 2), and their use in DME may be theoretically more rationale and comprehensive [20] than that of anti-VEGF agents that target only a part of the inflammatory cascade [51].

Animal experiments and clinical studies on DME patients have shown that IV injections of CSs are able to reduce the aqueous and vitreal levels of several pro-inflammatory chemokines and cytokines, including VEGF, whereas anti-VEGF agents decrease the VEGF concentration but do not alter the levels of other inflammatory molecules [77]. These findings can explain the persistence of DME despite repeated anti-VEGF IV injections [53,59,60,61].

### 4.2. Intravitreal Corticosteroids Used for the Treatment of DME

Intravitreal injection represents the most common route for the CSs’ ophthalmic administration in DME management, allowing rapid delivery of a large volume of drugs immediately available to the target site and limiting possible systemic side effects. Sustained-release CS implants have been developed in order to reduce the need for frequent IV injections [20,21].

Intravitreal CSs used to treat DME include triamcinolone acetonide (TA), dexamethasone (DEX), and fluocinolone acetonide (FAc) [20,21]. These three molecules have different receptor affinity, solubility, pharmacokinetic, and different gene regulation patterns, with consequently different clinical effects and safety characteristics [20,21].

**a**  **Tiamcinolone acetonide (TA)** [46]: This is commercially available as Kenalog-40 (Bristol-Myers Squibb, New York, NY, USA) or Tajoftal (Sooft Italia s.p.a. Montegiorgio, Udine, Italy), which is a crystalline powder available as an injectable suspension containing 40 mg/mL TA in isotonic saline solution and is delivered using a 30-gauge needle. TA is not approved for intraocular use, but it is used off-label to treat vitreoretinal diseases in a dose ranging between 1 and 4 mg [78], with functional and anatomical efficacy within 3–6 months post-injection [79]. Two other administration routes of TA tested in DME eyes, the posterior sub-tenon injection of 20 or 40 mg of TA [80] and the suprachoroidal injection of 2 or 4 mg of TA [81], have shown results comparable to those obtained with the IV TA injections, with less side effects. There is a preservative-free TA approved for intraocular use, though it is not for DME per se and not easily available, Triesence (Alcon Pharmaceuticals, Ft. Worth, TX, USA).
**b**  **Dexamethasone (DEX)** [42]: This is commercially available as a sustained-release biodegradable insert, the Ozurdex intravitreal implant (Allergan, Dublin, Ireland), which is a cylindrical tube (6 mm × 0.46 mm) composed of polylactic-co-glycolic acid polymers containing 0.7 mg of DEX, degrading into carbon dioxide and water as DEX is released into the vitreous body [82]. Ozurdex is delivered into the vitreous cavity using a single-use applicator with a 22-gauge needle for IV injection, and it was projected to endure a continuous IV release of micronized DEX over a period of ≤6 months. The peak of the functional and anatomical efficacy of the Ozurdex insert is typically reached at 2 months post-injection and has a duration of action of approximately 6 months [82].

The 3-year, randomized, multicenter, masked, sham-controlled clinical trial MEAD study led to the approval of the DEX 0.7 mg implant (Ozurdex) [42]. The FDA approved the Ozurdex IV implant for the treatment of adult patients with DME in 2014; the EMA approved Ozurdex in 2014 for the treatment of adult patients with visual impairment due to DME, retina vein occlusion, and noninfectious posterior segment uveitis, who are pseudophakic or who are considered insufficiently responsive or unsuitable for non-corticosteroid therapy [14]. The official product label in Europe recommends re-treatment of Ozurdex after approximately 6 months, and it does not recommend simultaneous administration in both eyes.

**c**  **Fluocinolone acetonide (FAc)** [40]: This is commercially available as an Iluvien^TM^ IV implant (Alimera Sciences Inc., Alpharetta, GA, USA), which is a sustained-release non-biodegradable IV insert containing 0.19 mg of FAc. Iluvien is a cylindrical tube (3.5 mm × 0.37 mm) of polymer loaded with FAc that is inserted into the vitreous cavity through a 25-gauge needle and releases 0.2 µg/day of FAc. The FAMOUS study has demonstrated that the Iluvien IV implant is able to maintain a therapeutic concentration of FAc over a period of 36 months [83]. The peak of the functional and anatomical efficacy of the Iluvien IV insert has been observed between 6 and 11 months post-injection [84,85]. Being a non-biodegradable implant, floaters have been complained about by some patients after Iluvien implant [84,85].

The Fluocinolone Acetonide in diabetic Macular Edema (FAME) study [40], a 3-year, randomized, sham injection-controlled, double-masked, multicenter clinical trial, led to the FDA approval, in 2014, of the FAc 0.19 mg IV implant (Iluvien^TM^) for the treatment of DME in patients who were previously treated with steroids and did not have a clinically significant IOP elevation, excluding patients with confirmed or suspected ocular or periocular infections, patients with glaucoma and CRD ≥ 0.8, and patients with known hypersensitivity to any component of the implant [14,84].

In 2014, the EMA approved the Iluvien IV implant for the treatment of vision impairment associated with non-infectious uveitis or chronic DME insufficiently responsive to other available therapies [14,84].

The official product label in Europe recommends retreatment after at least 1 year and does not recommend the simultaneous treatment of both eyes.

### 4.3. Pharmacology of Intravitreal Corticosteroids Used for the Treatment of DME

**a**  **Water solubility:** DEX is the one that is the most water soluble, which implies increased bioavailability but rapid elimination. For these reasons, it is available commercially as a sustained-release biodegradable implant [21]. Fac is 50% less water soluble than DEX but still requires a sustained-release delivery system to maintain an efficient IV concentration of the drug over time. TA has low water solubility, and it is available as an IV injectable suspension [21]. Sustained IV inserts have the advantage of reducing the frequency of IV injections, with a subsequent decrease in complications related to repeated IV injection procedures, higher patient compliance, and lower healthcare costs [21].
**b**  **Intravitreal pharmacokinetics**: Human studies analyzing aqueous humor samplings have demonstrated that the 4 mg TA IV injection has a mean elimination half-time of 15.4 ±1.9 days [86].

Animal studies (monkeys and rabbits) have shown that the Ozurdex IV implant has the highest rate of drug release during the first 2 months, followed by a prolonged lower level of release, with IV DEX levels not more detectable 6 months after the implant [82]. Moreover, DEX was detected in the plasma only in a small percentage of samples (12%) [82], suggesting a high systemic safety profile of the Ozurdex IV insert.

Human studies have demonstrated that, after the Iluvien IV implant, FAc was detectable in the aqueous humor at 36 months, and that the plasma levels of FA were always below the limits of quantification [83].

In comparison with both IV TA injection and FAc implant, Ozurdex IV insert has been demonstrated to provide extremely higher doses of steroids delivered into the vitreous body and the retina during the first 2 months of therapy. Conversely, the Iluvien insert was projected to release a sustained low concentration of steroids for a long period of time. Studies in rabbits have calculated that the vitreous maximum concentration after the IV injection of 4 mg TA, DEX 0.7 mg, and FAc 0.59 mg implants were 460 ng/mL, 1300 ng/mL, and 18 ng/mL, respectively [21]. The Ozurdex implant represents, therefore, a pulse administration of a high dose of CSs, which is a therapeutic modality successfully used to treat important inflammatory or autoimmune diseases, such as acute optic neuritis [67,68]. Moreover, the well-known phenomenon of reduced responsiveness to steroid treatment over time, likely related to the downregulation of the glucocorticoid receptors, is alleviated by a pulse dosing of CSs, as provided by the Ozurdex insert [21].

### 4.4. Clinical Efficacy and Safety Profile of Intravitreal Corticosteroids Used in DME Treatment

**a**  **Triamcinolone acetonide (TA)**

The studies investigating the efficacy and safety of the IV triamcinolone acetonide (IVTA) injections are summarized in Table A1.

RCTs failed to demonstrate the non-inferiority of IVTA in comparison with IV sham injections [87], macular laser photocoagulation [46], or anti-VEGF agents [59] so TA did not receive approval for DME treatment.

In particular, the DRCR.net Protocol B, a 3-year RCT including CIDME 840 eyes randomized to receive IV injections of TA 1 mg or 4 mg or focal/grid laser photocoagulation, demonstrated that IVTA was associated with lower VA gain and higher risk of IOP rise and cataract development than laser [46].

The DRCR.net Protocol I, a 5-year RCT including CIDME 854 eyes randomized to receive sham injection + prompt laser, 4 mg IVTA injection + prompt laser, or ranibizumab injection + prompt or deferred laser, showed that the VA gain was significantly higher in both ranibizumab groups (comparable between ranibizumab and TA in pseudophakic eyes), with lower local side effects [59].

IVTA has been widely used off-label with different dosages and intervals between administrations, showing to be an effective and relatively inexpensive method for DME management [88].

TA intravitreal injection has shown a clear time-limited therapeutic effect, with clinical efficacy for approximately 3 months [87,88,89,90].

The cumulative incidence of OHT after IVTA injections ranges between 13% and 50% [46,59,87,88,89,90,91,92,93]. The time required for the IOP elevation after a TA injection is 1–8 weeks, IOP reaches the peak value in 2–16 weeks, remains elevated for 1–9 months, and returns to pre-treatment values after 4–9 months [94]. The majority of cases of steroid-induced IOP rise post-IVTA (95–97% of cases) can be managed with ocular hypotensive drugs, whereas a minority of cases should receive glaucoma surgery [59,74,87].

The incidence of cataract extraction requirement after IVTA injections ranges between 10% and 83%, with a higher incidence in younger patients [46,59,87,88,89,90,91,92,93].

As compared to the anti-VEGF agents, IVTA provided lower functional results when both phakic and pseudophakic eyes were considered [59,91,92,93,95], and there was similar VA gain in pseudophakic eyes, where the confounding factor of the cataract development is excluded [59]. The morphological outcomes were comparable or lower than those related to the anti-VEGF agents [59,91,93,96].

The functional and anatomical outcomes of the association of IVTA with macular laser [97] or with anti-VEGF [96,98] were comparable to those obtained with macular laser or anti-VEGF as monotherapy and were linked to a higher risk of IOP rise and cataract development.

In conclusion, although IVTA has shown to be effective in DME management, its short duration of action and the high incidence of IOP elevation and cataract development, especially in younger patients, have limited its use in favor of other approved intravitreal CSs. More recently, TA administered as supra-choroidal [81,99] and sub-tenon injections [80] in DME eyes has shown promising although time-limited results.

**b**  **Dexamethasone**

RCTs and real-life studies investigating the therapeutic and side effects of the IV dexamethasone implant are reported in Table A2.

The registration study “MEAD” [42] was a 3-year, randomized, multicenter, masked, sham-controlled clinical trial including 1048 CIDME eyes (25% eyes were treatment-naïve) randomized to receive 0.7 mg or 0.35 mg DEX implant or sham procedure. Re-treatment was allowed no more often than every 6 months. The study demonstrated that both 0.7 mg and 0.35 mg DEX implants were significantly more effective than sham in improving VA and decreasing macular edema, although approximately 25% of eyes developed an IOP, 1.5% required glaucoma surgery, and 50–60% of phakic eyes required cataract surgery. The MEAD study demonstrated that the high-dose DEX insert provided the best benefit/risk ratio and led to the approval of the DEX 0.7 mg implant (Ozurdex) [42].

Another RCT [100] and numerous real-world studies [101,102,103,104] have demonstrated the effectiveness of the Ozurdex IV implant in DME treatment.

Published studies about Ozurdex implants having at least 24–36 months of follow-up reported a BCVA gain ranging between +2.8 and +9.6 letters [42,103,105,106]. The BCVA improvement was higher in pseudophakic eyes, where the confounding factor of the cataract development could be excluded [42,107,108]. The long-term functional response to Ozurdex seems to be predictable on the basis of the BCVA gain at 3 months post-injection [109]. Although the registration MEAD study allowed re-injections after 6 months, 35% of patients in the real-life studies required a re-injection between 3 and 5 months [101,110,111].

The Ozurdex IV implant has shown better functional and morphological results in treatment-naïve and in recent DME eyes [104,105,110,111,112], as well as when it was administered at need as opposed to a fixed regimen of 5 or 6 months of interval between re-injections [110,113]. The incidence of IOP elevation after the Ozurdex intravitreal implant ranges from 8% and 38% of cases [42,100,101,102,103,104,107,108,110,114,115,116,117]. Previous studies found that the IOP elevation after DEX implants was highly predictable, with the IOP peak occurring between 6 to 8 weeks post-op and returning to baseline values by around 3 to 4 months post-op [42,74,114,117]. Patients should, therefore, undergo a safety visit 6–8 weeks after the Ozurdex implant in order to evaluate the therapeutic response to the drug and to measure the IOP. Repeated injections of Ozurdex implants were not found to have any cumulative effect on the IOP [117].

The majority of cases of IOP elevation after the Ozurdex implant were generally transient and successfully managed with topical treatment, whereas filtration surgery was needed in 0–1.7% of cases [42,100,101,110,114,117], and the surgical options include trabeculectomy, shunts implant, and minimally invasive glaucoma surgery [118].

Cataract development or progression in phakic eyes after 0.7 mg DEX implant was 68% in the approval MEAD study [42], whereas real-life studies reported a lower incidence, ranging between 0% and 50%, due to the predominant selection of pseudophakic eyes [100,101,102,103,107,108,110,116,117,119]. The incidence of cataract development after Ozurdex varies depending on follow-up duration and type of treated pathology and seems to be directly correlated with the number of Ozudex implants [103,105,117].

Both RCTs and real-world studies showed that the Ozurdex implant is associated with high systemic safety, with systemic side effects involving less than 1% of patients, with the most frequent being hypertension worsening [42,110,117,120].

When compared to the macular focal/grid laser photocoagulation for CIDME management, Ozurdex has shown comparable functional results and better anatomical outcomes [102].

In general, the comparison of the Ozurdex IV implant and anti-VEGF in DME eyes has shown that Ozurdex is associated with the following:

- lower BCVA gain [107,108,120], which became comparable to anti-VEGF when pseudophakic eyes were analyzed separately [107,108,115];
- better anatomical results, i.e., higher ability in reducing the macular edema, that did not translate directly into better BCVA improvements [107,115,120,121,122,123,124], which may be likely due to the cataract development;
- comparative better results in naïve than in non-naïve eyes, whereas the outcomes in the two groups appear similar in anti-VEGF studies [105];
- better results in chronic and persistent DME eyes and in eyes with moderate-severe DME (CRT > 410 microns) [124,125]
- better outcomes in real-life studies than in interventional studies [126]: the possibility of retreating at an earlier stage and the higher number of naïve (2/3 of treatment-naïve eyes in anti-VEGF studies vs. 1/5 in the Ozurdex studies), or short duration DME eyes with better baseline VA in the real-life studies, may explain these differences (on the other hand, systematic review and meta-analysis studies have demonstrated that IV anti-VEGF showed better anatomical and functional results in RCTs compared to the observational studies, likely because fewer injections are administered in the observation studies than those suggested by the RCTs [105,120,126], and VA gain seems to be strictly related to the number of the IV injections, at least during the first year of therapy [127,128]);
- higher risk of OHT, glaucoma, and cataracts and lower risk of serious systemic adverse events [107,108,115,119,120,123];
- lower number of required IV injections [105,107,108,120]: the comparison of the results of the MEAD study (Ozurdex pivotal study) [42] and RESTORE study (ranibizumab pivotal study) [129] showed that the VA gain observed in pseudophakic eyes receiving 0.7 mg DEX in the MEAD study (6 letters over 3 years with 4–5 implants) was comparable to that achieved in the RESTORE study by eyes receiving a mean of 7 ranibizumab injection/year
- lower treatment costs, including medications, OCT, FA, and surgical procedures. The global cost of a 1-year therapy with Ozurdex is approximately one-half of that with anti-VEGF, which is mainly related to the significantly lower frequency of IV injections, even when the costs of the cataract and glaucoma surgeries are added [130].

Switching from anti-VEGF therapy to Ozurdex in cases of persistent and unresponsive DME has shown to be helpful [116,131], providing better functional and anatomical results and higher cost-effectiveness in cases of “early switch”, i.e., after non-adequate response to 3-monthly anti-VEGF injections [116,132].

As compared with the anti-VEGF agents as monotherapy, the association therapy of Ozurdex plus anti-VEGF has provided a BCVA gain similar [115] or better in the presence of high levels of OCT inflammatory biomarkers [119], with better anatomical results, and higher risks of IOP elevation and cataract development [115,119].

**c**  **Fluocinolone acetonide**

Table A3 lists the studies concerning the clinical efficacy and side effects of the fluocinolone acetonide IV implant.

The registration study Fluocinolone Acetonide in diabetic Macular Edema (FAME) [40] was a 3-year, randomized, sham injection-controlled, double-masked, multicenter clinical trials including 956 patients with persistent DME despite macular laser (median duration of DME of 3 years) randomized to receive intravitreal inserts releasing 0.2 µg/day or 0.5 µg/day FAc or sham injection. Re-treatment was allowed no more often than every 12 months. FAc-treated eyes showed significantly higher VA gain and CRT reduction as compared with sham, with better results in chronic DME cases, although they were associated with an IOP rise in approximately 35% of eyes, needing glaucoma surgery in 4.8–8.1% of cases, and required cataract surgery in 40–50% of phakic eyes. The mean number of FAc re-treatments was 1.3 over 3 years. FAc-treated eyes required adjunctive therapies, such as laser, IVTA, or anti-VEGF intravitreal injections, in more than 50% of cases. The FAME study demonstrated that the low-dose FAc insert provided the best benefit/risk ratio as compared with the high-dose one and allowed for the FDA approval of the 0.2 µg/day (0.19 mg) FAc intravitreal implant (Iluvien ^TM^) [40].

The clinical efficacy of the Iluvien intravitreal insert has been demonstrated by other RCTs and several real-life observational studies [133,134,135,136,137,138,139,140,141] and confirmed by systematic reviews, meta-analyses, and expert panels [142,143,144].

Published studies with the FAc implant with at least 36 months of follow-up reported a VA gain ranging between 3.6 and 11 letters [40,85,133,134,135,136,137,138,139,140,141].

Iluvien has shown higher effectiveness in chronic (>3 years duration) DME eyes [40] and has provided both functional and anatomic improvements in DME eyes with persistent DME and refractory to previous therapies with laser, anti-VEGF, or other intravitreal steroids (TA or DEX) [133,135,137,138,140,144,145,146].

Moreover, real-world studies with long follow-up showed that the long-active FAc implant was able to provide a long-lasting stabilization of the functional and, more importantly, of the anatomical outcomes, with decreased CRT variation for up to 3 years [134,136,138,141]. These results are crucial because reduced anatomical fluctuations have been associated with better functional improvements, whereas greater macular thickness variability in DME patients has been linked with neural damage and poorer visual outcomes [62,63].

Furthermore, with scheduled follow-ups every 4 months during the 36 months post-implant and a mean of 1.1 insert/3 years, Iluvien required a significantly lower frequency of treatment and check-up visits as compared with both anti-VEGF and other intravitreal steroids (TA and DEX), with a significant saving of time for diabetic patients, who are frequently pluri-medicated and poor-compliant, and a saving of costs for the public health system [147].

In steroid-responders, the IOP increase after the FA implant generally occurs within 2–4 weeks, reaches the peak at 24–48 weeks and returns to baseline values 9–12 months after implantation [74].

The approval study FAME reported that the 0.2 µg/d FAc insert was associated with an IOP rise ≥ 10 mmHg and the need for glaucoma surgery in 34% and 4.8% of cases, respectively (FAME). A post hoc analysis of the FAME study demonstrated that all glaucoma surgeries occurred in eyes with no history of a steroid challenge before the FAc implant [148], so that the FDA approved the IV FAc insert “in patients who have been previously treated with a course of corticosteroids and did not have a clinically significant rise in intraocular pressure”.

In real-world observational studies, the percentages of IOP-related side effects attributable to Iluvien appeared significantly lower than those reported by the FAME pivotal study [85]. Iluvien intravitreal implant was associated with a mean risk of post-operative IOP elevation of 20%, depending on the different definitions of IOP rise, with a risk of steroid-induced glaucoma of 0–10% [133,134,135,136,137,138,139,140,141,142,143,144,149] and a mean requirement of glaucoma surgeries of 0.6%, ranging from 0% to 4.3% [142,143], whereas a minority of cases should receive glaucoma surgery, including trabeculectomy, shunts implant, and minimally invasive glaucoma surgery [148].

This discrepancy between clinical trials and real-world data can be explained considering that, in clinical practice, almost only patients previously treated with intravitreal steroids without post-operative IOP elevation were selected [136,137,139,141].

Cataract development after 0.2 µg/d FAc implant was 82% in the FAME study, whereas in real-world studies, the incidence is limited by the prevalent selection of pseudophakic eyes, ranging between 40–65% of cases [133,134,135,136,137,138,139,140,141,142,143,144]. Phakic patients should expect to have cataract surgery planned between 13 and 18 months post-FAc injection [85].

Previous studies have shown that, although a single 3-year FAc implant was used in more than two-thirds of cases [40,142,143], additional treatments, such as laser photocoagulation, anti-VEGF, or intravitreal steroids achieving an acutely higher concentration, such as the TA or DEX implant, are required in the 33–75% of cases during the 3-year duration of the FAc implant [40,136,138,139,140,141,142,144]. On the other hand, when the FAc IV insert is considered as an additional therapy, it has been shown to significantly reduce the need for IV pharmacotherapy in DME eyes [85,134,141]. These supplementary treatments may represent a limitation and confounding factor in the evaluation of the Iluvien IV implant efficacy.

More recently, the suprachoroidal delivery of fluocinolone acetonide (Iluvien^R^) implant in eyes with chronic DME has provided promising results in improving visual function and reducing the incidence of steroid-induced cataract and glaucoma [150].

### 4.5. When to Choose Intravitreal Corticosteroids for DME Treatment (Early Switch from Anti-VEGF or First-Line Therapy)

Subgroups of DME patients who can benefit from therapy with intravitreal steroids should include the following:

**a**  **Patients unresponsive to anti-VEGF therapy:** Previous RCTs [53,59,60,61] have demonstrated that approximately 40–65% of patients showed persistent DME despite adequate anti-VEGF therapy. It is important to note that the current literature does not provide a univocal definition of “poor-response” or “non-response” to treatment, so highly heterogeneous guidelines on when and how to switch or stop the different therapeutic approaches in DME have been proposed. The most commonly used definition of persistent or refractory DME after intravitreal pharmacotherapies is a VA gain of <5 letters and/or a CST reduction of <20% on OCT as compared to baseline [14,16].

The reason for the heterogeneous response to the anti-VEGF therapy is not yet fully understood and may be related to differences in VEGF gene polymorphism and expression, patient age, glycemic control, DR severity, and DME duration [151]. Studies have shown that DME patients with high serum and aqueous humor levels of VEGF will show a good response to anti-VEGF, whereas patients with low to normal VEGF levels and higher levels of inflammatory biomarkers (Table 1) do not adequately respond to anti-VEGF treatments [152] and may likely benefit from IV CSs that have been demonstrated to modulate several inflammatory pathways.

Moreover, considering that the final response to anti-VEGF therapy seems to be predictable after 3 to 6 injections and to be independent of the number of injections [58,60], the evaluation of the response after 3–6 IV injections could be appropriate to decide to try an alternative therapy.

According to the current Euretina guidelines, intravitreal CSs are considered a second-line option restricted to anti-VEGF non-responders or patients who have reached a plateau (persistent DME and VA < 5/10) after 3–6 anti-VEGF injections, depending on the response of each single patient [14].

**b**  **Non-compliant patients or patients unable to maintain frequent follow-up visits:** DME patients are often working, aging, or in poor health, requiring, therefore, frequent health care visits, and may thus have difficulties adhering to frequent office visits or monthly injection protocols. Approximately 60% of DME patients are poor or noncompliant with intravitreal therapy [153].

Previous RCTs have shown that anti-VEGF therapy requires a rigorous injection schedule to provide favorable outcomes [41,43,60,61], with heavy burdens for both patients and caregivers, and the VA gain is directly related to the number of anti-VEGF injections, especially in the first years of treatment [128,154]. On the other hand, patients receiving anti-VEGF therapy in real-life studies are frequently undertreated because intensive treatment with anti-VEGF is harder to maintain in clinical practice than in clinical trials and reaches poor visual outcomes [105,126].

Non-compliant patients could, therefore, benefit from therapy with intravitreal slow-release steroids, aiming to avoid suboptimal visual function improvement because of a sub-dosing anti-VEGF therapy.

**c**  **Patients with recent arterial thromboembolism (ATE) events:** The relationship between IV anti-VEGF injections and the risk of ATEs is still debated [57], and it was not clarified by the majority of the RCTs in which patients with recent ATEs were excluded from the study [41,43,61]. On the other hand, real-world studies and systematic reviews have reported a link between anti-VEGF therapy and several systemic side effects, including deterioration of systemic hypertension, renal dysfunction, gastrointestinal perforation, stroke, myocardial infarction, and thromboembolic events [65,66].

As suggested by several international guidelines, in patients with DME and a history of stroke or myocardial infarction it could be more prudent to use intravitreal CSc as first-line treatment rather than anti-VEGF agents [13,14,15,16,17,18,19].

**d**  **Pregnant or breastfeeding women:** DME can progress rapidly during pregnancy, especially in DM type 1 [1,2,4], so the management in pregnancy could be a challenge. The use of anti-VEGF in pregnancy is not recommended because of its potential negative effects on the angiogenesis of developing embryos or fetuses, and several case series have demonstrated a correlation between anti-VEGF IV injections given at the first five weeks of gestation and miscarriages or pre-eclampsia [155]. Although a close observation is suggested in the majority of cases, focal laser photocoagulation or intravitreal Ozurdex implant should be considered as the first-line treatment for DME in pregnancy, when therapy is considered to be necessary [13,14,15,16,17,18,19].

Intravitreal TA [156] and DEX [157] have been already used in the treatment of DME in pregnancy, with no reported side effects.

**e**  **Presence of chronic DME:** In cases of chronic edema, anti-VEGF has demonstrated poor efficacy [60,151,158], whereas IV CSs have shown efficacy in persistent DME unresponsive to anti-VEGF therapy [40,42,89,125,133,135,137,138,140,144,145,146].

Previous studies have reported that Ozurdex induced a significant VA gain if administered in eyes with chronic DME resistant to anti-VEGF [124,125].

The Iluvien approval study FAME showed that FAc intravitreal implant was more effective in patients having DME for more than 3 years as compared to patients with more recent DME [40]. Moreover, in real-life studies, Iluvien has demonstrated the ability to increase BCVA and reduce macular thickness in DME patients with persistent DME after treatment with IV anti-VEGF and CSc (TA or Ozurdex) [133,135,137,138,140,144,145,146];

**f**  **Presence of hard exudates (HE) at the center of the fovea:** This represents a major complication of DME because it can cause severe central visual loss and it is a negative predictive factor for visual outcomes [5,6]. A post-hoc analysis of the RCT Bevordex study comparing Ozurdex implants every 4 months and monthly bevacizumab injections showed that Ozurdex was associated with greater regression of the HE at 12 months [108]. These results suggest the preferential use of DEX over anti-VEGF in eyes with foveal hard exudates.
**g**  **Associated OCT features of inflammation**: Many OCT features, biomarkers of a high level of retinal inflammation and/or chronic DME, seem to be able to predict a poor or suboptimal responsiveness to anti-VEGF treatment, suggesting, therefore, the use of CSs as first-line therapy or an early switch to CS-therapy [32], which include the presence of large intra-retinal para-foveal cysts, a CRT > 410 µm, a large extension of the disorganization of the inner and outer retinal layers, a higher amount of hyper-reflective foci (HRFs), and the presence of a sub-foveal serous retinal detachment (SRD) [32]. In particular, a greater level of HRF seems to be one of the most important predictors for a better response to CS than to anti-VEGF [159]. OCT biomarkers in DME are proposed in order to identify in general good or poor responders to various treatments and to guide the decision to switch to other treatment options, allowing for a more personalized treatment with better visual outcomes [32,160].
**h**  **Need for cataract surgery:** DM patients are at higher risk of developing or deteriorating DME after cataract surgery and have a higher incidence of post-surgery macular edema, the so-called Irvine–Gass syndrome (IGS) [161]. Previous authors have reported that 22% of diabetic patients and 30% of patients with DR developed or worsened DME within 1 year after cataract surgery [161]. The results of the anti-VEGF therapy in cases of post-cataract surgery DME or of IGS are inconclusive [162], whereas IV CSs have shown promising outcomes.

The off-label IV or sub-tenon administration of TA has been shown to prevent the development or increase of DME in diabetic patients after cataract surgery [163].

Ozurdex has been shown to be effective in preventing the onset or deterioration of DME and the IGS post-cataract surgery in diabetic patients when administered 2–4 weeks before, concurrently, or post-cataract surgery [164,165].

**i**  **Vitrectomized eyes:** The vitreous body serves as a reservoir of IV-injected drugs. The study of the efficacy of the anti-VEGF agents in vitrectomized eyes has been proven to be time-limited [166], whereas TA [167,168], Ozurdex [169], and Iluvien [170] have been shown to be effective in vitrectomized eyes and can be considered the first choice in vitrectomized eyes in suitable cases.

### 4.6. When to Avoid Intravitreal Corticosteroids/Prefer Anti-VEGF Agents for DME Treatment

**a**  **History of glaucoma or OHT:** IOP rise and glaucoma are the most frequent and important side effects of the IV CSs. An IOP elevation (IOP > 25 mmHg or IOP rise ≥ 10 mmHg) has been reported in 13–50% of cases with IVTA [46,59,94], in 8–38% after Ozurdex [114,117], and in 8–34% after Iluvien [138,141].

Patients with a higher risk of developing IOP elevation after IV steroid therapy are those with OHT, glaucoma, or previous steroid-associated IOP elevation [74,76].

For patients receiving IVTA, the topical CSs challenge had a positive predictive value of 100% and a negative predictive value of 60% [171], so that the utility of the topical CS challenge before the CS intravitreal administration of a different type of CS remains unclear [74].

Considering the Ozurdex IV implant, significant risk factors for post-injection IOP elevation have been demonstrated to be younger age, male sex, type 1 DM, history of uveitis, or preexisting glaucoma treated with two or three hypotensive agents [114]. In particular, glaucomatous patients treated with one, two, or three ocular hypotensive agents had, respectively, 37%, 50%, and 100% risk of being high CS-responders, i.e., of developing an IOP elevation of >15 mmHg after the first Ozurdex injection [114].

Based on the results of the approval FAME study [40], Iluvien has been approved by the FDA explicitly for the treatment of patients who were non-steroid-responders [14,16,17]. Indeed, the absence of IOP elevation after a prior steroid IV injection has been shown to have a positive predictive value ranging between 80% and 100% for a very low risk of IOP increase after the Iluvien implant [134,141,149], whereas eyes showing IOP elevation after Ozurdex had a 20-fold increased risk of developing an OHT after the Iluvien implant [137].

In consideration of all these clinical data, following the current guidelines, IVCS is not indicated in cases of advanced glaucoma treated with two or more anti-glaucomatous agents, and it is allowed in cases of OHT or early to moderate stable glaucoma well controlled with mono-therapy [14,16,17].

On the other hand, anti-VEGF therapy has been associated with an increased risk of persistent IOP rise requiring IOP-lowering treatment in only 5–10% of cases [66] and should be used as first-line DME treatment, especially in OHY and glaucomatous patients.

**b**  **Phakic patients with transparent crystalline:** The intravitreal CS treatment has been associated with a high rate of cataract development or progression, particularly in the second year of treatment [40,42,46,59,87,110,117,141,142,143,144]. The incidence of cataract development or progression has been reported to range between 10% and 83% for TA [46,59,87], between 0% and 68% for DEX [42,110,117], and between 40% and 82% for FAc [40,141,142,143,144], whereas it was between 0 and 15.4% after anti-VEGF injections in real-life studies [66].

Considering the cataractogenic effects of IV steroids, they are not suggested in children and young adults and subjects with transparent crystalline affected by DME.

**c**  **History of active or past ocular and periocular infections such as herpes or toxoplasmosis** [16]. The CSs have a strong immunosuppressive action, so they can exacerbate all types of infections, and case reports of reactivation of ocular herpetic infection [70] or of acute retinal necrosis [75] after Ozurdex IV implant have been described.
**d**  **Aphakia**, **absence or interruption of the posterior capsule**, **large iridectomy:** All these conditions are associated with the risk of the implant migration into the anterior chamber, which has been described both for Ozurdex [172] and for Iluvien [173]. The migration of the implant into the anterior chamber can lead to localized or diffuse corneal edema due to endothelial cell loss, which may be related to the chemical toxicity of the implant components or to the mechanical trauma of the rigid device in contact with the cornea [172,173].
**e**  **Presence of DME associated with advanced DR or with PDR:** Previous studies have shown that both IV anti-VEGF (ranibizumab and aflibercept) [53,54] and IV CSs (TA, Ozurdex, and Iluvien) [46,174,175] used for the treatment of DME were able to simultaneously reduce the risk of DR progression and PDR development. The five-year outcomes of the Bevordex study showed that patients receiving anti-VEGF (bevacizumab) were less likely to develop PDR than those receiving Ozurdex [108], suggesting that anti-VEGF may be superior to CSs because of its greater anti-angiogenic effect. Anti-VEGF agents are currently considered the first-line treatment option in eyes with DME associated with PDR [13,14,15,16,17,18,19].

### 4.7. Summary of the Efficacy and Safety of the Intravitreal Corticosteroid in DME Management

**a**  **Global efficacy and safety profile of intravitreal steroids:** The Cochrane Library systematic review published in 2020, including 10 RCTs (4505 eyes) and evaluating the efficacy and safety of IV steroids (TA, DEX, and FAc) as monotherapy for the treatment of DME, concluded that “IV steroids probably are more effective than sham treatment or control, with levels of evidence higher for FAc, lower for DEX and lowest for TA, providing small VA gain (1 ≤ Snellen line) in most studies; they probably are less effective than anti-VEGF in improving BCVA; they are associated with increased risk of cataract development and progression (20% in the control groups and 50–60% in the steroid groups), and may be therefore indicated in pseudophakic eyes; they are associated with IOP elevation (5% in control groups and 30% in steroids groups), need of IOP-lowering medications (1% in control groups and 33% in steroids groups), and need of glaucoma surgery (<1% of controls and 2% in patients treated with steroids); the need of glaucoma surgery is probably higher with FAc)” [176].
**b**  **Comparison of the efficacy profile amongst TA**, **DEX and FAc:** A direct comparison amongst TA, DEX, and FA has not yet been performed either in RCTs nor in large real-life studies. Moreover, having a different pharmacokinetic, they should probably not simply be compared but instead utilized in different selected cases. TA and DEX can be considered as an attack treatment, because, once injected, they immediately release an important dose of drug into the vitreal cavity [21]. On the other hand, the FAc implant can be defined as a background therapy because it delivers low concentrations of FAc into the eye for approximately 36 months [21]. The FAc implant aims to stabilize DME and should be injected preferably in DME eyes that respond to steroid therapy [85].

The “CONSTANT analysis” study has compared the effectiveness of Ozurdex and Iluvien for the treatment of DME found in their pivotal clinical trials and the MEAD [42] and FAME study [40], respectively, by calculating the area under the curve provided by the average letters gained across the entire treatment period (3 years) [177]. The results showed that, as compared with Ozurdex, Iluvien provided better long-term VA outcomes (5.2 vs. 3.5 letters/day, respectively) and a higher reduction of the CRT, with a lower treatment burden. Possible limitations of the MEAD study are that patients could be retreated with the Ozurdex no more often than every 6 months [42] and that cataract surgeries were performed with delay, between months 18 and 30, whereas in the FAME study, the median time of cataract extraction was 18 months [40].

Considering the results of the real-life studies, a small single-center retrospective study comparing the efficacy of Ozurdex and TA 2 mg in eyes with persistent DME after anti-VEGF treatment showed similar functional and anatomical results in the two groups [178]. Moreover, both Ozurdex and Iluvien have been shown to be effective in treating DME in patients previously unsuccessfully treated with TA [145,179]. Finally, the switch from Ozurdex to Iluvien has provided good functional and morphological results in chronic refractory DME eyes [137,139].

**c**  **Comparison of the safety profile amongst TA**, **DEX**, **and FAc:** The risk for steroid-induced OHT or SIG seems to be higher for FA, lower for TA, and lowest for DEX [74]. Furthermore, Ozurdex [42] is associated with a lesser risk of cataract development as compared with IVTA [46] or Iluvien [40]. These differences may be related to their different gene regulation pattern at the level of the human trabecular meshwork cell lines; moreover, DEX is less lipophilic than TA and FA, with less accumulation in the trabecular meshwork and lens, explaining the reduced incidence of IOP elevation and cataract with Ozurdex [180].

Finally, compared with other CS, DEX is associated with fewer systemic side effects [74].

**d**  **Comparison between intravitreal steroids and anti-VEGF:** The review of the literature suggests that both IV anti-VEGF agents and steroids are effective in DME treatment, although further pieces of evidence are needed to determine the comparative efficacies of these treatments [37,38].

Previous studies have shown that intravitreal CSs (TA and DEX) can provide similar VA improvements as anti-VEGF therapy, at least in pseudophakic eyes, where cataract progression does not limit functional performances [59,107,108,115]. A recent systematic review and meta-analysis, including 138 real-life observational studies representing more than 40,000 DME eyes treated with IV pharmacological agents or laser in the last decade, found that these therapies led generally to VA gain in real-world practice, with comparable results for anti-VEGF and CS (mean VA gain at 12 months of +4.6 letters for anti-VEGf and +4.4 letters for steroids) and significantly lower results associated with laser (+2.1 letters at 1-year follow-up), and that the clinical outcomes of the IV pharmacotherapies were significantly less impressive than those obtained in the RCTs, which was likely due to under-treatment and study population characteristics [106].

Other authors have recently underlined that both IV CSs and anti-VEGF do not result in a completely dry macula in approximately 50% of cases and that, because of their different mechanisms of action, the response can be better with one treatment compared to the other due to the disease and patient characteristics [181].

A new comprehensive review and meta-analysis conducted by the American Academy of Ophthalmology investigating efficacy and safety of the IV pharmacological therapy for DME reported that both anti-VEGF and CSs are similarly effective for DME treatment, with higher ocular side effects (cataract and IOP elevation) associated with CSs, especially in predisposed patients [182].

As already underlined, IV steroids are associate with high risk of IOP rise (0–35%) and cataract development (0–80%) [40,42,46,59,87,88,89,92], whereas these complications affect ≤ 15% of anti-VEGF-treated eyes [66].

On the other hand, IV CSs have shown high systemic safety, with ATEs incidence comparable to that found in controls [40,42,59], whereas IV anti-VEGF agents have been associated with significantly increased risk of systemic side effects and ATEs [65,66].

Moreover, IV CSs have shown to be more effective than anti-VEGF in cases of chronic and persistent or recurrent DME [40,42,124,125,133,135,137,138,140,144,145,146], where anti-VEGF have demonstrated poor efficacy in these cases [60,151,158].

Finally, as compared with anti-VEGF therapy, IV sustained-release CSs are associated with a significantly lower number of IV injections and check-up visits [107,108,134,141], which may reduce the injection-related complications [64] and improve patient compliance and reduce the costs for the public health system [130,147].

**e**  **Rationale of the association of intravitreal steroids and anti-VEGF agents:** These drugs have different mechanisms of action and could theoretically work well in combination. A systematic review and meta-analysis of the Cochrane Library published in 2018, which included 8 RCTs for a total of 817 eyes (the majority of which using bevacizumab plus TA), showed that the combination of IV anti-VEGF and steroids does not appear to provide additional visual benefit compared to monotherapy, exposing the patients to the potential side effects of both agents, such as cataract, glaucoma, stroke, and heart attack [183].

## 5. Guidelines for the Management of DME

Although metabolic control remains the most important strategy, a variety of therapeutic approaches is currently available for DME management, including laser photocoagulation, IV anti-VEGF or steroids, pars plana vitrectomy, or a combination of these therapies. Due to the multifactorial origin of DME, some patients may respond better to different therapeutic approaches.

DME type, severity and duration, features of the associated DR, ocular factors including lens status, IOP, history or presence of intraocular infections, associated comorbidities, such as presence of cardiovascular risk factors, overall patient compliance, recognition of predictive biomarkers, and identification of the response to treatment may guide the choice of the therapeutic strategy in order to personalize therapy to every single patient.

Following the current guidelines, IV pharmacotherapies with anti-VEGF agents or steroids are considered to be the first-line treatment in CIDME [13,14,15,16,17,18,19,37,38].

Both IV anti-VEGF and steroids have been shown to be effective in providing functional and morphological improvement in DME eyes [37,38]; steroids are associated with a higher risk of glaucoma and cataract development [21,73,74], and they are indicated in selected patients for this reason [184].

The current EURETINA Guidelines for the management of DME, published in 2017, suggest considering the use of intravitreal CS in “patients lacking a response to anti-VEGF agents or in patients in which the anti-VEGF therapy is contraindicated” and underline that “steroids maintain a role in the management of chronically persistent DME” [14].

Triamcinolone causes more IOP elevation and cataracts than Ozurdex and Iluvien, and it has not been approved for DME treatment [46,59]. It should be considered in cases that cannot obtain agents approved for DME [13,14,15,16,17,18,19].

Ozurdex may be an effective and safe second-line approach in patients with chronic persistent DME unresponsive to anti-VEGF agents [124,125], and a timely switch to an Ozurdex implant is essential in order to avoid irreversible loss of retinal cells due to persistent macular edema [116].

Ozurdex may be recommended as the first choice in specific DME patients, especially those who are pseudophakic, non-glaucomatous, and non-steroid-responders, including the following: patients with a history or at high risk of cardio- or cerebrovascular diseases; patients who are reluctant to receive frequent IV injections; patients that should undergo cataract surgery in the near future; vitrectomized patients; patients having chronic or severe macular edema; and patients with specific inflammatory biomarkers on OCT images [13,14,15,16,17,18,19].

An Iluvien implant could be considered as a second-third-line therapeutic alternative and it is particularly appropriate in pseudophakic patients, not CS-responders after Ozurdex or TA injection, with chronic DME, poor responders to anti-VEGF and Ozurdex, or good responders to anti-VEGF or Ozurdex but having poor compliance or unsatisfactory re-injection interval [84,85,143,149].

Moreover, Iluvien has been associated with a reduction of macular thickness fluctuations [134,136,138,141], which is linked to lower neural damage and better functional improvement [62,63].

Macular laser photocoagulation may play a role in non-CIDME for the treatment of leaking microaneurisms or capillaries [14,16]; moreover, it is particularly useful in developing countries, where the access to IV pharmacotherapies is limited, or when these therapies are contraindicated, such as during pregnancy or breastfeeding [13,14,15,16,17,18,19].

PPV in DME patients is indicated in the presence of vitreomacular tractions [13,14,15,16,17,18,19]. Other alternatives are the association of DEX or FAc implants with anti-VEGF, vitrectomy with peeling of the inner limiting membrane or epiretinal membrane if present, the addition of an FA exam in order to identify vascular anomalies or ischemic areas that need laser photocoagulation, or re-evaluation of the management of the systemic risk factors with a multidisciplinary team [85].

New therapeutic strategies acting on the inflammatory cascade or targeting neuroprotection in DME are actually under investigation in preclinical and clinical trials [11,12,15,185], including the following: agents binding other VEGF isoforms; port delivery systems with anti-VEGF; gene therapy to deliver anti-VEGF agents; inhibitors of the VEGF-receptors; agents targeting pro-inflammatory molecules such as neuropilin-1, integrin, TNF-alfa, interleukines, vascular adhesion protein-1, ang-2/tyrosin kinase; and molecules modulating the reactivity of the macro- and microglial cells. Neuroprotective agents have been also used for DME treatment, including erythropoietin (EPO), cibinetide, somatostatin, and brimonidine. All these neuroprotective agents failed to show significant clinical efficacy in DME patients and were not approved for DMR treatment.

## 6. Conclusions

Due to its multifactorial pathophysiology, the treatment of DME is complex, frequently requires a multidisciplinary approach, and still represents a clinical challenge considering that a substantial percentage of patients show a suboptimal response to treatments resulting in persistent visual loss [181]. Intravitreal pharmacotherapy with anti-VEGF or steroids is considered the first-line treatment in DME patients [37,38], whereas macular laser photocoagulation and pars plana vitrectomy may be useful in selected cases. Both IV anti-VEGF agents and steroids have shown efficacy in improving functional and morphological parameters in CIDME eyes, even if further pieces of evidence are needed in order to assess the comparative efficacy of these therapeutic approaches [37,38].

CSs represent an alternative therapeutic strategy in DME because of their well-known multiple immunosuppressive, anti-inflammatory, and anti-angiogenic effects [20,21], and their use may be more rationale and comprehensive [20] than anti-VEGF agents that target only a part of the inflammatory cascade [51].

RCTs and real-life studies have demonstrated that, in comparison with steroids, anti-VEGF agents are associated with higher retinal thickness fluctuations [41,43,53,54], require a more demanding treatment regimen with greater patient compliance [128], and have a lower systemic safety profile [65,66]. Conversely, steroids can reach a higher stabilization of the retinal morphology with a lower number of treatments [85,105] but are associated with a lower local safety profile, with a higher risk of glaucoma and cataract development [73,74]. Patients should, therefore, be clearly informed about the possible development of IOP elevation, glaucoma, or cataracts before obtaining their consent to treatment with IV steroids.

The optimization of the efficacy and safety of the treatment of DME with steroids requires a precise patient selection. Ideal candidates are pseudophakic patients, non-steroid-responders, without ocular hypertension or glaucoma, with low compliance, or at high risk of thromboembolic events. Alternative therapeutic strategies targeting inflammation, oxidative stress, and neurodegeneration have been proposed, but none of them have currently been approved [185]. A better elucidation of the complex pathogenic mechanism of DME will lead to the identification of new targets and therapeutic strategies for DME therapy.

## Figures and Tables

**Table 1:** Inflammatory mediators involved in DME pathogenesis.

vascular endothelial growth factor (VEGF)-A
angiopoietin-2 (Ang-2)
tumor necrosis factor (TNF)-α
placental growth factor (PGF)
hepatocyte growth factor (HGF)
insulin-like growth factor (IGF)
platelet activation factor (PAF)
interleukines (IL) IL-1, IL-6, IL-8
reactive oxygen species (ROS)
nitric oxide (NO)
protein kinase C (PKC)
advanced glycation products (AGEs)
monocyte chemoattractant protein-1 (MCP-1)
nitric oxide synthase (iNOS)
matrix metalloproteinases (MMPs)
intercellular adhesion molecule-1 (ICAM-1)
kallikrein-kinins (KKs)
chemokines
prostaglandins
leukotrienes
histamine
complement factors
activated retinal microglia
activated macrophages, lymphocytes, and neutrophyles

**Table 2:** Anti-inflammatory and anti-angiogenic properties of intra-vitreal corticosteroids.

Down-regulation of VEGF-A by reducing the expression of the VEGF-A genes and by regulating the expression of the VEGF-A receptors
Down-regulation of several other pro-inflammatory mediators, including interleukin-6, intercellular adhesion molecule-1 (ICAM-1), P-selectine, nitric oxide (NO), prostaglandins, and leukotrienes
Inhibition of leukostasis, with significant reduction of the neutrophil, lymphocyte, and macrophage migration through the blood vessel walls to the inflammation tissue sites
Restoration of the structural integrity of the tight junctions of the blood-retinal barriers
Inhibition of the collagenase with reduction of the vascular permeability
Downregulation of the expression of the aquaporin-4 in the Mueller cells’ membrane, thus reducing the intracellular edema

**Figure A1:**
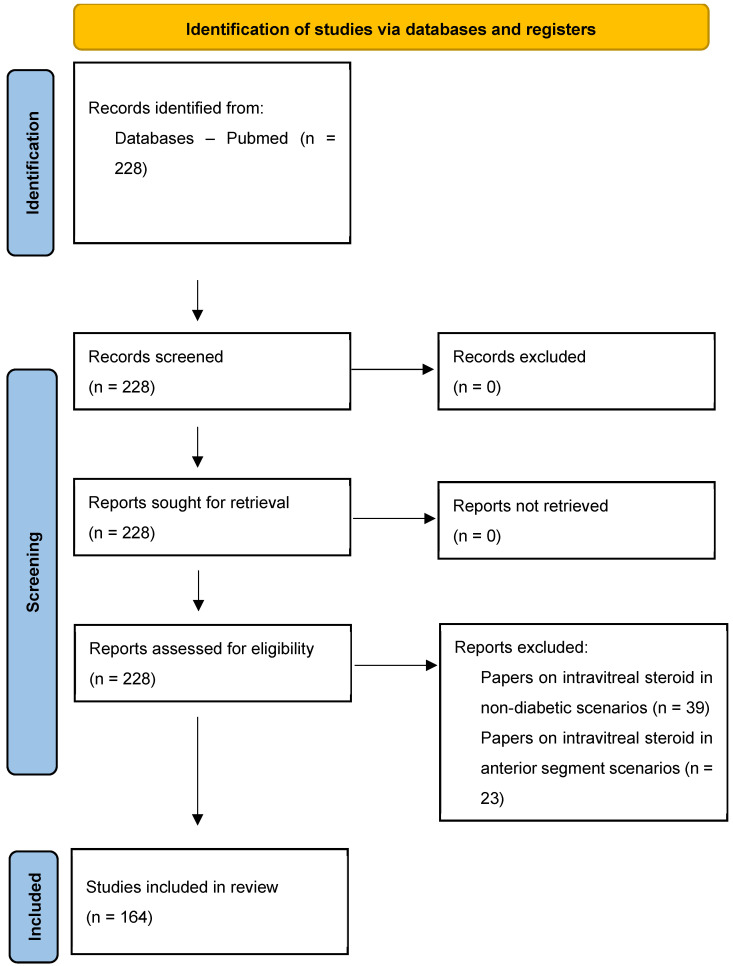
PRISMA flowchart showing search methodology.

## Data Availability

Not applicable.

## Abbreviations

DM	diabetes mellitus
DR	diabetic retionopathy
DME	diabetic macular edema
CSc	corticosteroids
TA	triamcinolone acetonide
DEX	dexamethasone
FAc	fluocinolone acetonide
VEGF	vascular endothelial growth factors
PPV	pars plana vitrectomy
IOP	intraocular pressure
BRB	blood-retinal barriers
RPE	retinal pigment epithelium
CSME	Clinically Significant Macula Edema
RCT	randomized controlled trial
ETDRS	Early Treatment of Diabetic Retinopathy Study
CIDME	center-involved-DME
VA	visual acuity
BCVA	best corrected visual acuity
DRCR.net	Diabetic Retinopathy Clinical Research.Network
FAG	fluorescein angiography
OCT	optical coherence tomography
CRT	central retinal thickness

## Appendix A

**Table A1:** Efficacy and safety of triamcinolone acetonide intravitreal injection.

Authors	Treatments	Study Type	Population	Study Focus	Follow-Up	Efficacy	Side Effects	Others
Beck et al., 2009 (DRCR.net Protocol B) [46]	IVTA 1 mg and 4 mg; focal/gris macular laser	RCT	840 CIDME eyes (BCVA 20/40–20/230)	Randomized treatment with 1 mg or 4 mg IVTA or focal/grid macular laser	3 years	Higher VA gain with macular laser as compared to both IVTA groups	In laser, 1-mg and 4-mg IVTA groups, IOP rise (>10 mmHg) in 4%, 18%, and 33% of eyes; cataract surgery needed in 31%, 46%, and 83% of eyes, respectively	IVTA-treated eyes associated with lower rate of DR progression: laser linked with higher risk of macular laser scars and subretinal fibrosis
Gillies et al., 2009 [87]	IVTA 4 mg; IV sham	RCT	69 CIDME eyes	Randomized treatment with 4 mg IVTA or sham, with adjunctive laser treatment where appropriate	5 years	VA gain and CRT reduction slightly but not significantly higher with IVTA	45% of the phakic eyes need cataract surgery in IVTA group	Comparable % of eyes requiring laser treatment in the two groups
Yilmaz et al., 2009 [79]	IVTA or sub-tenon TA	Systematic review of 6 RCTs	CIDME eyes refractory to laser	IVTA vs. no treatment or vs. TA sub-tenon injections	6 months	Significant VA gain and CRT reduction with IVTA until month 3, but not at month 6	Significantly higher IOP in both TA groups at month 3 and 6	
Elman et al., 2010 (DRCR.net Protocol I) [59]	IVTA 4 mg; IV sham; macular laser; IV ranibizumab	RCT	854 CIDME eyes (BCVA 20/32–20/320)	Randomized treatment with IV sham + prompt laser, 4 mg IVTA + prompt laser or IV ranibizumab + prompt or deferred laser	5 years	VA gain higher in both ranibizumab groups, and comparable between ranibizumab and TA groups in pseudophakic eyes. CRT reduction similar in TA and ranibizumab groups, and lower in sham group.	As compared with the other groups, TA eyes had IOP elevation (IOP rise ≥ 10 mmHg or IOP > 30 mmHg) in 50% vs. 10%; cataract development in 60% vs. 14%.	
Soheilian et al., 2012 [96]	IVTA 4 mg or IV bevacizumab	RCT	150 CIDME	Randomized treatment with IV bevacizumab or IV bevacizumab + IVTA or grid/focal laser every 3 months as needed	2 years	Better VA gain in the bevacizumab group at 6 months, comparable results amongst the 3 groups at 2 years; similar CRT reduction		
Kriechbaum et al., 2013 [95]	IVTA 8 mg	RCT	30 CIDME treatment-naïve eyes	Randomized treatment with 1 IVTA or IV 3 monthly bevacizumab with re-treatment at need	1 year	BCVA gain higher in the bevacizumab group; CRT reduction similar between groups	Higher cataract development in the TA group	
Zajac-Pytrus et al., 2017 [89]	IVTA 20 mg	Prospective interventional study	110 CIDME eyes treatment-naïve or unresponsive to laser	Evaluation of efficacy and safety of IVTA	6 months	Significant VA gain and CRT reduction until month 3, but not at month 6	IOP rise at month 1 and 3	
Arain et al., 2018 [98]	IVTA; IV bevacizumab	Prospective interventional study	50 pseudophakic eyes with refractory CIDME	Evaluation of efficacy and safety of IVTA + IV bevacizumab	3 months	Significant VA gain and CRT reduction until month 3		
Ogura et al., 2019 [80]	Sub-tenon 20 mg TA or 40 mg TA	RCT	95 CIDME eyes	Evaluation of efficacy and safety of sub-tenon TA 20 mg or 40 mg	3 months	Significant VA gain and CRT reduction in both groups until month 3	At month 3, IOP rise in 9% and 13%, and cataract development in 6% and 10% of eyes in the 20-mg TA and 40-mg TA, respectively	
Rodrigues et al., 2020 [91]	IVTA 4 mg; IV bevacizumab	RCT	65 CIDME unresppnsive to 6 monthly IV bevacizumab	Randomized treatment with IVTA 4 mg or IV bevacizumab at need	1 year	BCVA stable in the bevacizumab group and reduced in the TA group; similar CRT reduction in both groups	Higher risk of IOP rise in TA group	
Abdel-Maboud 2021 [92]	IVTA; IV bevacizumab	Systematic review and meta-analysis of 17 RCTs	1243 CIDME eyes	Randomized treatment with IVTA or IV bevacizumab	12 months	Higher BCVA gain and CRT reduction with bevacizumab in comparison with TA or TA + bevacizumab	Higher risk of IOP rise with TA	
Barakat et al., 2021 [99]	Suprachoroidal TA; IV aflibercept	RCT	71 treatment-naïve CIDME eyes	Randomized treatment with suprachoroidal TA + IV aflibercept every 3 months or monthly IV aflibercept intravitreal injections	6 months	Similar VA gain, better CRT reduction in the combination group	Higher local side effect (IOP rise and cataract) in the combination group	Lower number of treatments in the combination group
Sorrentino et al., 2021 [90]	IVTA 4 mg	Prospective, interventional, non-comparative real-life	49 CIDME eyes	Evaluation of efficacy and safety of IVTA	6 months	Significant VA gain and CRT reduction until month 6	Moderate IOP rise until month 3, with baseline values at 6 months	
Zhang et al., 2021 [97]	IVTA 4 mg; macular laser	Systematic review and meta-analysis of 8 studies (CRT and real-world studies)	549 CIDME eyes	Comparison of IVTA, macular laser and TA + laser	1 year	The early-term effect of IVTA + laser similar to IVTA alone but superior to laser alone; the long-term effect of IVTA + laser is similar to IVTA alone or laser alone	Higher risk of cataract and IOP rise associated with IVTA + laser o IVTA alone	Better effect if IVTA is administered before laser treatment
Nawar, 2022 [81]	Suprachoroidal TA 4 mg	Prospective nonrandomized interventional	55 eyes with CIDME refractory to anti-VEGF	Evaluation of efficacy and safety of suprachoroidal TA	12 months	Significant VA gain and CRT reduction until 12 months	Significant IOP increase at month 1, which returned to baseline values at month 3	Baseline BCVA and OCT morphology of intra-retinal and outer-retinal layers are predictors of the final BCVA
Zhu et al., 2022 [93]	IVTA 4 mg	Prospective RCT	102 CIDME eyes	Randomized treatment with IVTA or IV aflibercept	6 months	Higher VA gain and CRT decrease in the aflibercept group	Higher risk of local side effects in the TA group	Humor aqueous level od VEGF and other inflammatory mediators lower in the aflibercept group

TA = triamcinolone acetonide; IV = intravitreal; CIDME = center-involved-diabetic macular edema; VA = visual acuity; BCVA = best corrected visual acuity; CRT = central retinal thickness; RCT = randomized controlled trial; DRCR.net = Diabetic Retinopathy Clinical Research.Network; VEGF = vascular endothelial growth factor.

**Table A2:** Efficacy and safety of Dexamethasone intravitreal implant in diabetic macular edema.

Authors	Treatments	Study Type	Population	Study Design	Follow-Up	Efficacy	Side Effects	Others
Callanan et al., 2013 (PLACID study) [100]	0.7 mg DEX IV implant (Ozurdex) or macular laser	Multicenter RCT	253 CIDME eyes	Randomized treatment with sham implant + laser or 0.7 mg DEX IV implant + laser	1 year	higher VA gain at month 1 and 9 with DEX + laser, comparable between groups at 12 months; better morphological outcomes in the DEX + laser group.	16% vs. 1.6% of eyes required IOP-lowering medication; amongst phakic eyes, 22% vs. 9.5% developed cataract in the DEX + laser and sham + laser groups, respectively.	
Boyer et al., 2014 (MEAD study, registration study of the 0.7 mg DEX intravitreal implant, Ozurdex) [42]	0.35 mg or 0.7 mg DEX IV implant; sham IV injection	Multicenter RCT	1048 CIDME with BCVA of 20/50–20/200 and CRT ≥ 300 µm 25% of eyes were treatment -naïve	randomized treatment with 0.7 mg or 0.35 mg DEX IV implant or sham procedure Re-treatment no more often than every 6 months	3 years	Percentage of eyes with ≥15 letters BCVA gain of 22%, 18% and 12% respectively in DEX 0.7-mg, DEX 0.35-mg and sham group; significant CRT reduction in both DEX groups; severe systemic side effects <1% in both DEX and sham groups	IOP rise ≥ 10 mmHg in 28%, 25% and 4% respectively in DEX 0.7-mg, DEX 0.35-mg and sham group; glaucoma surgery in 1.5%, 0.9% and 0.3%; cataract development in 68%, 64% and 20% of phakic eyes, respectively	DEX groups were associated with significant decrease of aqueous inflammatory mediators; mean number of DEX re-treatment: 4/3 years
Lam et al., 2015 (CHROME study) [101]	0.7 mg DEX IV implant (Ozurdex)	Multicentre retrospective observational real-life	120 eyes with ME of at least 1 year (DME, RVO, uveitis)	Evaluation of efficacy and safety of 0.7 mg DEX IV implant	≥3 months	Significant VA increase and CRT decrease	IOP rise ≥ 10 mmHg in 21% of eyes; glaucoma surgery in 1.7% of eyes; cataract surgery in 30% of phakic eyes	Re-injection interval time of 2.3–4.9 months
Cornish et al., 2016 (BEVORDEX study) [108]	0.7 mg DEX IV implant (Ozurdex); bevacizumab IV injection	RCT	68 CIDME eyes	Randomized treatment with Ozurdex implant every 4 months or monthy bevacizumab IV injections as needed	2 years	Better functional results in bevacizumab group, similar between DEX and bevacizumab in pseudophakic eyes; similar anatomical outcomes	22% of DEX eyes and 0% of eyes treated with bevacizumab needed IOP-lowering drugs; 37% of DEX group and 6% in the bevacizumab group underwent cataract surgery	Mean number of injections lower in DEX group (2.8 vs. 9.1)
Shah et al., 2016 [121]	0.7 mg DEX IV implant (Ozurdex); bevacizumab IV injection	RCT	50 eyes with persistent CIDME despite ≥ 3 IV anti-VEGF injections	Randomized treatment with 1 Ozurdex implant or 3 monthy bevacizumab IV injections	7 months	Similar VA gain; greater CRT reduction in the Ozurdex group	Need of IOP-lowering medication greater in the Ozurdex group	
Heng et al., 2016 (OZLASE study) [102]	0.7 mg DEX IV implant (Ozurdex); macular laser	RCT	80 CIDME eyes	Randomized treatment with repeated Ozurdex + laser or macular laser	1 year	VA gain only in the laser group; CRT decrease only in the Ozurdex + laser group	Need of IOP-lowering drugs in 20% of eyes in the Ozurdex + laser groups and 2.5% of eyes in the laser group; 33% of phakic eyes in the Ozurdex + laser group required cataract surgery	
Al-Khersan et al., 2017 [109]	0.7 mg DEX IV implant (Ozurdex)	Multicentre retrospective review of real-life studies	102 CIDME eyes	Comparison of long-term functional results between eyes with poor (VA gain < 5 letters) and robust response (VA gain ≥ 10 letters) at 3 months	≥18 months	Early treatment functional response is directly correlated with the overall change in BCVA	n.a.	
Callanan et al., 2017 [107]	0.7 mg DEX IV implant (Ozurdex); bevacizumab IV injection	Multicenter RCT	163 persistent CIDME eyes with VA of 20/200 and 20/40 and CRT ≥ 300µm	Randomized treatment with Ozurdex implant every 5 months or monthy bevacizumab intravitreal injections as needed	1 year	Higher VA gain in the bevacizumab group, similar between groups in pseudophakic eyes; better anatomical outcomes with Ozurdex;	IOP elevation respectively in 34.3% of eyes in the DEX group, and 1.6% in the ranibizumab group; cataract development in 9.4% in the DEX group, and 0% in the ranibizumab group.	Mean number of injections lower with Ozurdex (2.8 vs. 8.5 in a year)
Malcles et al., 2017 (Reldex study) [103]	0.7 mg DEX IV implant (Ozurdex)	Prospective observational real-life	128 DME eyes	Evaluation of efficacy and safety of 0.7 mg DEX IV implant	3 years	Mean BCVA increase of +9.5 letters; significant CRT decrease	IOP ≥ 25 mmHg in 10% of eyes; cataract surgery in 47% of phakic eyes	mean of 3.6 DEX injections/3 years
Malcles et al., 2017 (SAFODEX study) [114]	0.7 mg DEX IV implant (Ozurdex)	Retrospective observational real-life	421 ME eyes (DME in 30% of cases, RVO, uveitis)	Evaluation of the safety profile (risk of IOP rise and glaucoma) of Ozurdex IV insert	3–55 months	n.a.	IOP ≥ 25 mmHg in 20% of eyes; need of IOP-lowering medication in 30% of eyes; glaucoma surgery in 0.4% of cases; risk factors for IOP rise: pre-existing glaucoma treated with 2 or 3 hypotensive drugs	Glaucomatous patients treated with one, two or three hypotensive agents had respectively 37%, 50% and 100% of high responders (i.e IOP rise > 15 mmHg)
Sarao et al., 2017 [113]	0.7 mg DEX IV implant (Ozurdex)	Multicenter RCT	42 CIDME eyes	Randomized treatment with Ozurdex administration at need or single Ozurdex administration	6 months	Better functional and anatomical results in the Ozurdex at need treatment group	Comparable safety profile between groups	Mean number of treatments: 1.6/6 months in the Ozurdex at need group
Bucolo et al., 2018 [110]	0.7 mg DEX IV implant (Ozurdex)	Systematic review of real-world studies	21 studies (831 DME eyes)	Comparison of Ozurdex IV implant every 6 months or at need	5–23 months	Better functional and anatomical results with implant at need, with mean time of re-treatment of 4–5 months	IOP rise in 15–30% of eyes, no need of glaucoma surgery; cataract development in 10–50% of cases	1/3 of the eyes were re-treated before six months from the first Ozurdex injection; mean re-treatment time of 5.3 ± 0.9 months; direct relationship between number of re-treatments and DME duration
Busch et al., 2018 [131]	0.7 mg DEX IV implant (Ozurdex); anti-VEGF IV injection	Multicentre retrospective case-control	110 eyes with DME unresponsive to anti-VEGF	Comparison of efficacy of the switch to Ozurdex after 3 monthly anti-VEGF injection or the prosecution of anti-VEGF therapy	1 year	VA change of −0.4 letters in the anti-VEGF group and +6.1 letters in the DEX group; CRT change of +18 µm in the anti-VEGF eyes and −93 µm in the DEX eyes	n.a.	
He et al., 2018 [120]	0.7 mg DEX IV implant (Ozurdex); anti-VEGF IV injection	Meta-analysis of RCTs	4 RCTs (521 DME eyes)	Comparison of efficacy and safety of Ozurdex intravitreal implant vs. anti-VEGF intravitreal injections	1 year	Ozurdex provided better anatomical outcomes despite significantly lower functional results (cataract development)	Ozurdex was associated with significantly fewer IV injections, higher risk of IOP elevation and cataract development, and lower risk of serious systemic adverse events	Treatment-naive eyes 17% in Ozurdex studies and 2/3 in anti-VEGF studies
Kodjikian et al., 2018 [105]	0.7 mg DEX IV implant (Ozurdex); anti-VEGF IV injection	Systematic review and meta-analysis of real-life studies	32 studies on anti-VEGF (6842 DME eyes) and 31 studies on Ozurdex IV implant (1703 eyes)	Evaluation of efficacy of Ozurdex IV implant and anti-VEGF IV injections	6–48 months	Higher VA gain with Ozurdex (mean of +9.6 letters with Ozurdex and +4.4 with anti-VEGF) Better results in naïve eyes with Ozurdex, similar results in naïve and non-naïve with anti-VEGF	n.a.	Mean number of injections per year: 1.6 with Ozurdex and 5.8 with anti-VEGF
Maturi et al., 2018 (DRCR.net Protocol U) [115]	0.7 mg DEX IV implant (Ozurdex); ranibizumab IV injections	RCT	129 eyes with CIDME refractory to ranibizumab IV injections	Randomized treatment to Ozurdex + ranibizumab or sham + ranibizumab after 3 monthly ranibizumab injections	2 years	Similar VA gain between groups; higher VA gain in the Ozurdex + ranibizumab in pseudophakic eyes; better morphological results in the Ozurdex + ranibizumab group	Need of IOP-lowering drugs in 30% of eyes in Ozurdex + ranibizumab group and in 0% in sham + ranibizumab group	
Castro-Navarro et al., 2019 [111]	0.7 mg DEX IV implant (Ozurdex)	Retrospective observational real-life	29 treatment-naïve and 55 non-naïve CIDME eyes	Comparison of efficacy of 0.7 mg DEX IV implant between naïve and refractory DME eyes	6 months	Better functional results in naïve eyes		35% of eyes required re-injection between months 3 and 6 post-op
Martinez et al., 2019 [116]	0.7 mg DEX IV implant (Ozurdex)	Retrospective observational real-life	69 eyes with CIDME unresponsive to anti-VEGF	Comparison of the efficacy and safety of the switch to Ozurdex after 3 (early switch) or 6 monthly anti-VEGF injections (late switch)	2 years	Better functional and anatomical results in the “early-switch” group	IOP rise in 10% and 26% of early- and late-switch groups respectively; 10% of phakic eyes required cataract extraction	Mean number of Ozurdex implants: 1.1/2 years in the early- and 1.7/2 years in the late-switch group
Rosenblatt et al., 2020 (European DME Registry Study) [104]	0.7 mg DEX IV implant (Ozurdex)	Multicentre retrospective Observational real-life	340 naïve-treatment and non-naïve DME eyes	Evaluation of efficacy and safety of 0.7 mg DEX intravitreal implant	6 months	VA gain of ≥15 letters in 23%, VA gain of ≥10 letters in 38% and VA lost of ≥15 letters in 8% of eyes. Better functional results in naïve eyes, with less DME duration and better glycemic control	IOP rise of >25 mmHg in 8% of eyes	The peak of improvement was achieved 3 months after the injection and dissipated until 6 months
Rajesh et al., 2020 (International Ozurdex Study Group) [117]	0.7 mg DEX IV implant (Ozurdex)	Multicenter retrospective observational real-life	2736 ME eyes (DME in 52% of cases, RVO, uveitis)	Evaluation of the safety profile of Ozurdex insert	6–102 months	n.a.	IOP > 25 mmHg in 26% of eyes; 0.5% required glaucoma surgery; cataract development in 47% of cases; 32.5% of eyes required cataract surgery; endophthalmitis, retinal detachment, and vitreous hemorrhages < 0.1% of eyes	
Zarranz-Ventura et al., 2020 [112]	0.7 mg DEX IV implant (Ozurdex)	Retrospective observational real-life	203 naïve-treatment and non-naïve DME eyes	Comparison of efficacy and safety of 0.7 mg DEX IV implant between treatment-naïve and non-naïve DME eyes	2 years	Naïve eyes had better functional and anatomical results, with lower number of re-injections and longer time to re-treatment		
Comet et al., 2021 (INVICTUS study) [122]	0.7 mg DEX IV implant (Ozurdex); ranibizumab IV injection; aflibercept IV injection	Prospective non randomized observation real-life study	70 treatment-naïve CIDME eyes	Comparison of efficacy of Ozurdex IV implant, ranibizumab or aflibercept IV injection	1 year	Similar functional results amogst groups; better anatomical outcomes with Ozurdex	n.a.	
Veritti et al., 2021 [126]	0.7 mg DEX IV implant (Ozurdex); anti-VDEGF IV injection	Systematic review and meta-analysis of RCTs and real-life studies	72 studies (45,032 DME eyes)	Evaluation and comparison of efficacy and safety of Ozurdex IV implant vs. anti-VEGF IV injections	12 months	VA gain obtained using anti-VEGF agents slightly but not significantly higher in RCTs than observational studies;Ozurdex implant showed significantly better results in observational studies than in RCTs.		With both RCTs and real-life studies, higher functional results with aflibercept as compared to Ozurdex and bevacizumab (similar to ranibizumab); with observational studies only, VA gain similar between anti-VEGF and Ozurdex
Gascon 2022 (INVICOST study) [130]	0.7 mg DEX IV implant (Ozurdex); ranibizumab IV injection; aflibercept IV injection	Prospective observational	60 treatment-naïve DME eyes	Comparison of the costs of the treatment with Ozurdex, aflibercept and ranibizumab	1 year	Ozurdex was significantly less expensive than that with anti-VEGF agents (approximately one half), mostly because of the lower frequency of intravitreal injections	n.a.	
Chi et al., 2023 [124]	0.7 mg DEX IV implant (Ozurdex)	Systematic review and meta-analysis of RCTs and real-life studies	30 studies (10 RCTs, 3 prospective real-world studies and 17 retrospective real-world studies) 2409 CIDME eyes	Comparison of efficacy and safety of Ozurdex IV implant vs. anti-VEGF IV injections	1–12 months	Similar functional results in non-resistant or naïve DME eyes, and better results for Ozurdex in resistant DME eyes; anatomical results significantly better for Ozurdex in both resistant and non-resistant DME eyes;	Side effects were comparable between the two groups	
Kaya et al., 2023 [119]	0.7 mg DEX IV implant (Ozurdex); ranibizumab IV injection	Prospective, consecutive clinical interventional	68 treatment-naïve CIDME eyes with OCT inflammatory biomarkers	Randomized treatment to Ozurdex + ranibizumab or ranibizumab monotherapy	2 years	VA gain ≥15 letters in 65% and 26% of Ozurdex + ranibizumab and ranibizumab groups respectively; greater CRT decrease in the combination group;	IOP rise ≥5 mmHg and cataract development in 38% and 27% of the combination group, and in 18% and 12% of the ranibizumab monotherapy group	
Patil et al., 2023 [123]	0.7 mg DEX IV implant (Ozurdex); IVTA injection; anti-VEGF IV injection	Systematic review and meta-analysis of RCTs	14 RCTs (827 DME eyes)	Comparison of efficacy and safety of intravitreal anti-VEGF and steroids (Ozurdex or TA)	1 year	DEX and TA were associated with comparable functional results and significantly better anatomical outcomes	DEX and TA were associated with significantly higher risk of IOP elevation	
Ruiz-Moreno et al., 2023 [132]	0.7 mg DEX IV implant (Ozurdex)	Multicentre retrospective cost analysis	108 eyes with CIDME unresponsive to anti-VEGF	Evaluation of the cost-effectiveness of the switch to Ozurdex after 3 (early-switch) or >3 (late-switch) anti-VEGF injections	1 year	Early switch to Ozurdex is more cost-effective than late-switch		

DEX = dexamethasone; TA = triamcinolone acetonide; IV = intravitreal; CIDME = center-involving diabetic macular edema; VA = visual acuity; BCVA = best corrected visual acuity; CRT = central retinal thickness; RCT = randomized controlled trial; VDEGF = vascular endothelial growth factor; DRCR.net = Diabetic Retinopathy Clinical Research.Network; n.a. = not applicable.

**Table A3:** Efficacy and safety of fluocinolone acetonide intravitreal implant.

Authors	Drug	Study Type	Population	Study Focus	Follow-Up	Efficacy	Side Effects	Others
Campochiaro et al., 2012 (FAME study, registration study of the 0.2 µg/d FAc IV implant (Iluvien) [40]	0.2 µg/d or 0.5 µg/d FAc IV implant; IV sham	Multicenter RCT	956 eyes with persistent CIDME despite macular laser with BCVA of 20/50–20/400 and CRT ≥ 250 µm	Randomized treatment with intravitreal inserts releasing 0.2 µg/day or 0.5 µg/day FAc or sham injection.	3 years	VA gain ≥ 15 letters in 29%, 28% and 19% in the 0.5 µg/d, 0.2 µg/d and sham group; better results in chronic DME (3 years)	In the 0.5 µg/d FAc, 0.2 µg/d FAc and sham groups: IOP rise ≥ 10 mmHg in 37%, 34% and 10%; need of glaucoma surgery in 8.1%, 4.8% and 0.5%; cataract development in 89%, 82% and 27% of eyes.	Need of adjunctive therapies in 51%, 54% and 95% of the 0.5 µg/d FAc, 0.2 µg/d FAc and sham groups; mean number of re-treatments: 1.3/3 years; Need of glaucoma surgery only in steroid-responders
Peto 2017 (Iluvien Clinical Evidence Study (ICE-UK) [133]	0.2 µg/d FAc IV implant (Iluvien)	Multicentre retrospective observational real-world	233 eyes with chronic and refractory CIDME	Evaluation of efficacy and safety of Iluvien IV implant	1 year	Mean VA gain of +3.8 letters; mean CRT decrease of −113 µm	19% of eyes required IOP-lowering drugs; no need of glaucoma surgery	Significant VA gain only by eyes with baseline VA > 55 letters; eyes with CRT ≥ 400 µm at baseline were more likely to achieve e significant CRT reduction
Ch’ng et al., 2018 [147]	0.2 µg/d FAc IV implant (Iluvien)	Retrospective cost analysis	14 eyes with refractory CIDMEswitched from IV pharmacotherapy to Iluvien	Evaluation of costs before and after the switch to Iluvien	3 years	Switch to Iluvien is a cost-saving procedure	n.a.	Switch to Iluvien is a time-saving procedure
Eaton et al., 2019 (the USER study) [134]	0.2 µg/d FAc IV implant (Iluvien)	Retrospective chart review	160 CIDME eyes (91% non-treatment-naïve)	Evaluation of efficacy and safety of Iluvien IV implant	3 years	Stable VA for up to 3 years; CRT decrease; significant reduction of CRT fluctuation	IOP ≥ 25 mmHg in 31% of eyes; need of IOP-lowering medication in 24% of eyes; no need of glaucoma surgery	Reduction number of treatment (anti-VEGF or Ozurdex) from 1 every 2.9 months pre-FAc implant to 1 every 14.3 months after-FAc implant; non-steroid-responders to Ozurdex have a probability of 96% of being non-steroid responders to Iluvien
Rehak et al., 2019 [136]	0.2 µg/d FAc IVimplant (Iluvien); 0.7 mg DEX IV implant (Ozurdex)	Retrospective chart review	59 eyes with CIDME responsive to anti-VEGF and/or Ozurdex but recidivant after stopping treatment	Comparison between switching directly from anti-VEGF to Iluvien and indirectly via Ozurdex to Iluvien after >4 months	3 years	Significant VA gain similar in both groups (+10.6 and +9.7 letters); significant CRT reduction after 3 months of Iluvien implant and maintained for up to 36 months	IOP rise > 10 mmHg in 26% of eyes; cataract development in 73% of phakic eyes	37% of eyes required additional treatments
Augustin et al., 2020 (Retro-IDEAL study) [135]	0.2 µg/d FAc IV implant (Iluvien)	Retrospective observational real-world	81 CIDME eyes unresponsive to anti-VEGF	Evaluation of efficacy and safety of the switch from anti-VEGF to Iluvien IV implant	3 years	VA gain of +5.5 letters from month 9 and maintained for 3 years; CRT decrease	IOP rise in 27% of eyes managed with IOP-lowering medications	
Fallico et al., 2021 [142]	0.2 µg/d FAc IV implant (Iluvien)	Systematic review and meta-analysis of real-world studies	9 real-world studies (7 retrospective and 2 prospective) (428 eyes)	Evaluation of efficacy and safety of Iluvien IV implant	2 years	Significant VA gain and CRT decrease	Need of IOP-lowering drugs in 27% of eyes; need of glaucoma surgery in 3% of cases; cataract extraction in 39% of phakic eyes	Need of supplementary therapies in 39% of eyes
Kodjikian et al., 2021 [143]	0.2 µg/d Fac IV implant (Iluvien)	Systematic review of real-world studies	22 observational real-world studies (1880 DME eyes)	Evaluation of efficacy and safety of Iluvien IV implant	≥1 year	mean peak of VA gain of +8.7 letters 1 year post-op; mean CRT decrease of 34% from baseline	IOP rise 20% of eyes; 0.6% required glaucoma surgery; cataract extraction in 43% of phakic eyes	Higher VA gain associated with lower BCVA at baseline and more recent DME
Bailey et al., 2022 (Medisof audit study) [138]	0.2 µg/d FAc IV implant (Iluvien)	Multicenter retrospective observational real-world	256 eyes with chronic and refractory CIDME (89% psedophakic)	Evaluation of the long-term efficacy and safety of the Iluvien IV implant	4 years	VA gain ≥5, ≥10 letters and ≥15 letters in 46%, 25% and 17% of eyes respectively; stabilization of the retinal morphology for 4 years.	IOP increase ≥10 mmHg in 29%, use of IOP-lowering medications in 28% and glaucoma surgery in 2.7% of eyes	Mean number of FAc inserts: 1.1/4 years; need of IOP-lowering treatments in 50% of patients with prior history of OHT or glaucoma and 18% of those without
Baillif et al., 2022 [139]	0.2 µg/d FAc IV implant (Iluvien)	Multicenter, retrospective observational	113 CIDME responsive to Ozurdex without IOP rise	Evaluation of efficacy and safety of the switch from Ozurdex to Iluvien	1 year	Significant VA gain and CRT decrease	No side effects, IOP ≤ 18 mmHg during the entire follow-up	Need of additional treatments in 33% of eyes; shorter time between last Ozurdex and Iluvien implant (≤8 weeks) associated with reduced CRT fluctuations and reduced need for additional treatments
Cicinelli et al., 2022 [137]	0.2 µg/d FAc IV implant (Iluvien)	Retrospective observational real-life	54 chronic DME pseudophakic eyes previously treated with Ozurdex	Evaluation of efficacy and safety of the switch from Ozurdex to Iluvien	1 year	Significant VA gain and CRT reduction	>20-fold risk of IOP rise in eyes showing IOP-elevation post-Ozurdex	Direct correlation between morphological response to Ozurdex and Iluvien; absence of relationship between VA gain post-Ozurdex and post-Iluvien
Mathies et al., 2022 (REALFAc study) [140]	0.2 µg/d FAc IV implant (Iluvien)	Prospective observational real-life	62 eyes with chronic (mean DME duration of 60 months) refractory CIDME	Evaluation efficacy and safety of the Iluvien IV implant	1 year	VA gain ≥5 letters in 50% of eyes; CRT decrease;	Need of IOP-lowering medications in 18% of cases	Need of additional treatment in 37% of cases
Singer et al., 2022 (PALADIN study) [141]	0.2 µg/d FAc IV implant (Iluvien)	Prospective, multicentre, observational, non-randomized, open-label	202 non-steroid-responders CIDME eyes	Evaluation of the long-term efficacy and safety of the Iluvien IV implant	3 years	mean BCVA increase of +3.6 letters and mean CRT reduction of −60.7 µm, with reduced VA and CRT fluctuation for 3 years	IOP rise ≥10 mmHg in 27.7% of eyes; need of IOP-lowering drugs in 38% of eyes; glaucoma surgery in 1.5% of eyes; cataract surgery in 62% of phakic eyes	Eyes with IOP < 25 mmHg after local steroids had IOP < 25 mmHg after FAc in 97% of cases; need of additional therapy in 75% of eyes, with frequency decrease from 3.5/year before Iluvien to 1.7/year after Iluvien
Khoramnia et al., 2023 (ILUVIEN Registry Safety Study—IRISS) [144]	0.2 µg/d FAc IV implant (Iluvien)	Multicenter, open-label, observational real-world registry study	695 eyes with chronic CIDME	Evaluation of the long-term efficacy and safety of the Iluvien intravitreal implant	4 years	Mean VA gain of +5 letters; VA gain ≥15 letters in 18% of eyes	IOP elevation ≥ 10 mmHg in 15% of eyes; need of IOP-lowering medications in 35%; glaucoma surgery in 4.3%; cataract extraction.in 65% of the phakic eyes	Mean number of FAc inserts: 1.1/4 years; need of supplementary therapies in 44% of eyes
Roth et al., 2023 [149]	0.2 µg/d FAc IV implant (Iluvien)	Observational real-world study	202 CIDME eyes after a successful prior IV steroid challenge	Evaluation of the safety profile of the Iluvien IV implant	3 years	n.a.	IOP > 25 mmHg in 32% of eyes; IOP >30 mmHg in 16% of cases	Eyes with IOP < 25 mmHg after previous IV steroids had IOP < 25 mmHg after FAc in 78% of cases

FAc = fluocinolone acetonide; DEX = dexamethasone; IV = intravitreal; CIDME = center-involved-diabetic macular edema; VA = visual acuity; BCVA = best corrected visual acuity; CRT = central retinal thickness; RCT = randomized controlled trial; OHT = ocular hypertension; n.a. = not applicable.

## References

[1] Cloete L. (2021). Diabetes mellitus: An overview of the types, symptoms, complications and management. Nurs. Stand..

[2] Lovic D., Piperidou A., Zografou I., Grassos H., Pittaras A., Manolis A. (2020). The Growing Epidemic of Diabetes Mellitus. Curr. Vasc. Pharmacol..

[3] Riddle M.C., Herman W.H. (2018). The Cost of Diabetes Care—An Elephant in the Room. Diabetes Care.

[4] Lin K.-Y., Hsih W.-H., Lin Y.-B., Wen C.-Y., Chang T.-J. (2021). Update in the epidemiology, risk factors, screening, and treatment of diabetic retinopathy. J. Diabetes Investig..

[5] Holekamp N.M. (2016). Overview of diabetic macular edema. Am. J. Manag. Care.

[6] Bandello F., Battaglia Parodi M., Lanzetta P., Loewenstein A., Massin P., Menchini F., Veritti D. (2017). Diabetic Macular Edema. Dev. Ophthalmol..

[7] Yoshimura T., Sonoda K.-H., Sugahara M., Mochizuki Y., Enaida H., Oshima Y., Ueno A., Hata Y., Yoshida H., Ishibashi T. (2009). Comprehensive analysis of inflammatory immune mediators in vitreoretinal diseases. PLoS ONE.

[8] Klaassen I., Van Noorden C.J., Schlingemann R.O. (2013). Molecular basis of the inner blood-retinal barrier and its breakdown in diabetic macular edema and other pathological conditions. Prog. Retin. Eye Res..

[9] Romero-Aroca P., Baget-Bernaldiz M., Pareja-Rios A., Lopez-Galvez M., Navarro-Gil R., Verges R. (2016). Diabetic Macular Edema Pathophysiology: Vasogenic versus Inflammatory. J. Diabetes Res..

[10] Daruich A., Matet A., Moulin A., Kowalczuk L., Nicolas M., Sellam A., Rothschild P.-R., Omri S., Gélizé E., Jonet L. (2018). Mechanisms of macular edema: Beyond the surface. Prog. Retin. Eye Res..

[11] Starace V., Battista M., Brambati M., Cavalleri M., Bertuzzi F., Amato A., Lattanzio R., Bandello F., Cicinelli M.V. (2021). The role of inflammation and neurodegeneration in diabetic macular edema. Ther. Adv. Ophthalmol..

[12] Zhang J., Zhang J., Zhang C., Zhang J., Gu L., Luo D., Qiu Q. (2022). Diabetic Macular Edema: Current Understanding, Molecular Mechanisms and Therapeutic Implications. Cells.

[13] Puliafito C.A., Cousins S.W., Bacharach J., Gonzalez V.H., Holekamp N.M., Merrill P.T., Ohr M.P., Parrish R.K., Riemann C.D. (2016). Forming a Consensus: Data and Guidance for Physicians Treating Diabetic Macular Edema. Ophthalmic Surg. Lasers Imaging Retin..

[14] Schmidt-Erfurth U., Garcia-Arumi J., Bandello F., Berg K., Chakravarthy U., Gerendas B.S., Jonas J., Larsen M., Tadayoni R., Loewenstein A. (2017). Guidelines for the Management of Diabetic Macular Edema by the European Society of Retina Specialists (EURETINA). Ophthalmologica.

[15] Browning D.J., Stewart M.W., Lee C. (2018). Diabetic macular edema: Evidence-based management. Indian J. Ophthalmol..

[16] Kodjikian L., Bellocq D., Bandello F., Loewenstein A., Chakravarthy U., Koh A., Augustin A., de Smet M.D., Chhablani J., Tufail A. (2019). First-line treatment algorithm and guidelines in center-involving diabetic macular edema. Eur. J. Ophthalmol..

[17] Amoaku W.M., Ghanchi F., Bailey C., Banerjee S., Banerjee S., Downey L., Gale R., Hamilton R., Khunti K., Posner E. (2020). Diabetic retinopathy and diabetic macular oedema pathways and management: UK Consensus Working Group. Eye.

[18] Chhablani J., Wong K.M., Tan G.S.F., Sudhalkar A.M., Laude A.F., Cheung C.M.G.F.M., Zhao P.F., Uy H., Lim J., Valero S.M. (2020). Diabetic Macular Edema Management in Asian Population: Expert Panel Consensus Guidelines. Asia-Pac. J. Ophthalmol..

[19] Giridhar S., Verma L., Rajendran A., Bhend M., Goyal M., Ramasamy K., Rajalakshmi, Padmaja R., Natarajan S., Palanivelu M.S. (2021). Diabetic macular edema treatment guidelines in India: All India Ophthalmological Society Diabetic Retinopathy Task Force and Vitreoretinal Society of India consensus statement. Indian J. Ophthalmol..

[20] Zhang X., Wang N., Schachat A.P., Bao S., Gillies M. (2014). Glucocorticoids: Structure, signaling and molecular mechanisms in the treatment of diabetic retinopathy and diabetic macular edema. Curr. Mol. Med..

[21] Whitcup S.M., Cidlowski J.A., Csaky K.G., Ambati J. (2018). Pharmacology of Corticosteroids for Diabetic Macular Edema. Investig. Ophthalmol. Vis. Sci..

[22] Liberati A., Altman D.G., Tetzlaff J., Mulrow C., Gøtzsche P.C., Ioannidis J.P., Clarke M., Devereaux P.J., Kleijnen J., Moher D. (2009). The PRISMA statement for reporting systematic reviews and meta-analyses of studies that evaluate healthcare interventions: Explanation and elaboration. BMJ.

[23] (1985). Photocoagulation for diabetic macular edema. Early Treatment Diabetic Retinopathy Study report number 1. Early Treatment Diabetic Retinopathy Study research group. Arch. Ophthalmol..

[24] Klein R., Klein B.E., Moss S.E., Cruickshanks K.J. (1995). The Wisconsin Epidemiologic Study of Diabetic Retinopathy. XV. The long-term incidence of macular edema. Ophthalmology.

[25] Damaskos C., Garmpis N., Kollia P., Mitsiopoulos G., Barlampa D., Drosos A., Patsouras A., Gravvanis N., Antoniou V., Litos A. (2020). Assessing Cardiovascular Risk in Patients with Diabetes: An Update. Curr. Cardiol. Rev..

[26] Baker C.W., Glassman A.R., Beaulieu W.T., Antoszyk A.N., Browning D.J., Chalam K.V., Grover S., Jampol L.M., Jhaveri C.D., Melia M. (2019). Effect of Initial Management With Aflibercept vs. Laser Photocoagulation vs. Observation on Vision Loss Among Patients With Diabetic Macular Edema Involving the Center of the Macula and Good Visual Acuity: A Randomized Clinical Trial. JAMA.

[27] Murakami T., Yoshimura N. (2013). Structural changes in individual retinal layers in diabetic macular edema. J. Diabetes Res..

[28] Acón D., Wu L. (2018). Multimodal Imaging in Diabetic Macular Edema. Asia-Pac. J. Ophthalmol..

[29] Huang Z., Qiu K., Yi J., Lin H., Zheng D., Huang D., Zhang G., Chen H., Zheng J., Wang Y. (2022). Diabetic retinopathy with extensively large area of capillary non-perfusion: Characteristics and treatment outcomes. BMC Ophthalmol..

[30] Virgili G., Menchini F., Casazza G., Hogg R., Das R.R., Wang X., Michelessi M. (2015). Optical coherence tomography (OCT) for detection of macular oedema in patients with diabetic retinopathy. Cochrane Database Syst. Rev..

[31] Im J.H., Jin Y.-P., Chow R., Yan P. (2022). Prevalence of diabetic macular edema based on optical coherence tomography in people with diabetes: A systematic review and meta-analysis. Surv. Ophthalmol..

[32] Munk M.R., Somfai G.M., de Smet M.D., Donati G., Menke M.N., Garweg J.G., Ceklic L. (2022). The Role of Intravitreal Corticosteroids in the Treatment of DME: Predictive OCT Biomarkers. Int. J. Mol. Sci..

[33] Lee J., Moon B.G., Cho A.R., Yoon Y.H. (2016). Optical Coherence Tomography Angiography of DME and Its Association with Anti-VEGF Treatment Response. Ophthalmology.

[34] Tranos P.G., Wickremasinghe S.S., Stangos N.T., Topouzis F., Tsinopoulos I., Pavesio C.E. (2004). Macular edema. Surv. Ophthalmol..

[35] Apte R.S., Chen D.S., Ferrara N. (2019). VEGF in Signaling and Disease: Beyond Discovery and Development. Cell.

[36] Sohn E.H., van Dijk H.W., Jiao C., Kok P.H.B., Jeong W., Demirkaya N., Garmager A., Wit F., Kucukevcilioglu M., van Velthoven M.E.J. (2016). Retinal neurodegeneration may precede microvascular changes characteristic of diabetic retinopathy in diabetes mellitus. Proc. Natl. Acad. Sci. USA.

[37] Gurreri A., Pazzaglia A. (2021). Diabetic Macular Edema: State of Art and Intraocular Pharmacological Approaches. Adv. Exp. Med. Biol..

[38] Tatsumi T. (2023). Current Treatments for Diabetic Macular Edema. Int. J. Mol. Sci..

[39] Dugel P.U., Hillenkamp J., Sivaprasad S., Vögeler J., Mousseau M.-C., Wenzel A., Margaron P., Hashmonay R., Massin P. (2016). Baseline visual acuity strongly predicts visual acuity gain in patients with diabetic macular edema following anti-vascular endothelial growth factor treatment across trials. Clin. Ophthalmol..

[40] Campochiaro P.A., Brown D.M., Pearson A., Chen S., Boyer D., Ruiz-Moreno J., Garretson B., Gupta A., Hariprasad S.M., Bailey C. (2012). Sustained delivery fluocinolone acetonide vitreous inserts provide benefit for at least 3 years in patients with diabetic macular edema. Ophthalmology.

[41] Nguyen Q.D., Brown D.M., Marcus D.M., Boyer D.S., Patel S., Feiner L., Gibson A., Sy J., Rundle A.C., Hopkins J.J. (2012). Ranibizumab for diabetic macular edema: Results from 2 phase III randomized trials: RISE and RIDE. Ophthalmology.

[42] Boyer D.S., Yoon Y.H., Belfort R., Bandello F., Maturi R.K., Augustin A.J., Li X.-Y., Cui H., Hashad Y., Whitcup S.M. (2014). Three-Year, randomized, sham-controlled trial of dexamethasone intravitreal implant in patients with diabetic macular edema. Ophthalmology.

[43] Korobelnik J.-F., Do D.V., Schmidt-Erfurth U., Boyer D.S., Holz F.G., Heier J.S., Midena E., Kaiser P.K., Terasaki H., Marcus D.M. (2014). Intravitreal aflibercept for diabetic macular edema. Ophthalmology.

[44] Diabetes Control and Complications Trial Research Group (1995). Progression of retinopathy with intensive versus conventional treatment in the Diabetes Control and Complications Trial. Ophthalmology.

[45] UK Prospective Diabetes Study (UKPDS) Group (1998). Intensive blood-glucose control with sulphonylureas or insulin compared with conventional treatment and risk of complications in patients with type 2 diabetes (UKPDS 33). Lancet..

[46] Beck R.W., Edwards A.R., Aiello L.P., Bressler N.M., Ferris F., Glassman A.R., Hartnett E., Ip M.S., Kim J.E., Diabetic Retinopathy Clinical Research Network (DRCR.net) (2009). Three-year follow-up of a randomized trial comparing focal/grid photocoagulation and intravitreal triamcinolone for diabetic macular edema. Arch Ophthalmol..

[47] Sabal B., Teper S., Wylęgała E. (2022). Subthreshold Micropulse Laser for Diabetic Macular Edema: A Review. J. Clin. Med..

[48] Lois N., Campbell C., Waugh N., Azuara-Blanco A., Maredza M., Mistry H., McAuley D., Acharya N., Aslam T.M., Bailey C. (2023). Diabetic Macular Edema and Diode Subthreshold Micropulse Laser: A Randomized Double-Masked Noninferiority Clinical Trial. Ophthalmology.

[49] Jackson T.L., Nicod E., Angelis A., Grimaccia F., Pringle E., Kanavos P. (2017). Pars plana vitrectomy for diabetic macular edema: A Systematic Review, Meta-Analysis, and Synthesis of Safety Literature. Retina.

[50] Khattab A.A.A., Ahmed M.M., Hammed A.H. (2022). Pars plana vitrectomy for tractional diabetic macular edema with or without internal limiting membrane peeling. Med. Hypothesis Discov. Innov. Ophthalmol..

[51] Fogli S., Del Re M., Rofi E., Posarelli C., Figus M., Danesi R. (2018). Clinical pharmacology of intravitreal anti-VEGF drugs. Eye.

[52] Rajendram R., Fraser-Bell S., Kaines A., Michaelides M., Hamilton R.D., Degli Esposti S., Peto T., Egan C., Bunce C., Leslie R.D. (2012). A 2-year prospective randomized controlled trial of intravitreal bevacizumab or laser therapy (BOLT) in the management of diabetic macular edema: 24-month data: Report 3. Arch. Ophthalmol..

[53] Reddy R.K., Pieramici D.J., Gune S., Ghanekar A., Lu N., Quezada-Ruiz C., Baumal C.R. (2018). Efficacy of Ranibizumab in Eyes with Diabetic Macular Edema and Macular Nonperfusion in RIDE and RISE. Ophthalmology.

[54] Heier J.S., Korobelnik J.-F., Brown D.M., Schmidt-Erfurth U., Do D.V., Midena E., Boyer D.S., Terasaki H., Kaiser P.K., Marcus D.M. (2016). Intravitreal Aflibercept for Diabetic Macular Edema: 148-Week Results from the VISTA and VIVID Studies. Ophthalmology.

[55] Singh R.P., Barakat M.R., Ip M.S., Wykoff C.C., Eichenbaum D.A., Joshi S., Warrow D., Sheth V.S., Stefanickova J., Kim Y.S. (2023). Efficacy and Safety of Brolucizumab for Diabetic Macular Edema: The KINGFISHER Randomized Clinical Trial. JAMA Ophthalmol..

[56] Wykoff C.C., Abreu F., Adamis A.P., Basu K., Eichenbaum D.A., Haskova Z., Lin H., Loewenstein A., Mohan S., Pearce I.A. (2022). Efficacy, durability, and safety of intravitreal faricimab with extended dosing up to every 16 weeks in patients with diabetic macular oedema (YOSEMITE and RHINE): Two randomised, double-masked, phase 3 trials. Lancet.

[57] Virgili G., Parravano M., Evans J.R., Gordon I., Lucenteforte E. (2017). Anti-vascular endothelial growth factor for diabetic macular oedema: A network meta-analysis. Cochrane Database Syst. Rev..

[58] Gonzalez V.H., Campbell J., Holekamp N.M., Kiss S., Loewenstein A., Augustin A.J., Ma J., Ho A.C., Patel V., Whitcup S.M. (2016). Early and Long-Term Responses to Anti–Vascular Endothelial Growth Factor Therapy in Diabetic Macular Edema: Analysis of Protocol I Data. Am. J. Ophthalmol..

[59] Elman M.J., Aiello L.P., Beck R.W., Bressler N.M., Bressler S.B., Edwards A.R., Ferris F.L., Friedman S.M., Glassman A.R., Diabetic Retinopathy Clinical Research Network (2010). Randomized trial evaluating ranibizumab plus prompt or deferred laser or triamcinolone plus prompt laser for diabetic macular edema. Ophthalmology.

[60] Bressler N.M., Beaulieu W.T., Glassman A.R., Blinder K.J., Bressler S.B., Jampol L.M., Melia M., Wells J.A. (2018). Diabetic Retinopathy Clinical Research Network Persistent Macular Thickening Following Intravitreous Aflibercept, Bevacizumab, or Ranibizumab for Central-Involved Diabetic Macular Edema With Vision Impairment: A Secondary Analysis of a Randomized Clinical Trial. JAMA Ophthalmol..

[61] Glassman A.R., Wells J.A., Josic K., Maguire M.G., Antoszyk A.N., Baker C., Beaulieu W.T., Elman M.J., Jampol L.M., Sun J.K. (2020). Five-Year Outcomes after Initial Aflibercept, Bevacizumab, or Ranibizumab Treatment for Diabetic Macular Edema (Protocol T Extension Study). Ophthalmology.

[62] Starr M.R., Salabati M., Mahmoudzadeh R., Patel L.G., Ammar M.J., Hsu J., Garg S., Ho A.C., Kuriyan A.E. (2021). Fluctuations in Central Subfield Thickness Associated With Worse Visual Outcomes in Patients With Diabetic Macular Edema in Clinical Trial Setting. Am. J. Ophthalmol..

[63] Wang V.Y., Kuo B.L., Chen A.X., Wang K., Greenlee T.E., Conti T.F., Singh R.P. (2022). Fluctuations in macular thickness in patients with diabetic macular oedema treated with anti-vascular endothelial growth factor agents. Eye.

[64] Patel D., Patel S.N., Chaudhary V., Garg S.J. (2022). Complications of intravitreal injections: 2022. Curr. Opin. Ophthalmol..

[65] Porta M., Striglia E. (2020). Intravitreal anti-VEGF agents and cardiovascular risk. Intern. Emerg. Med..

[66] Zehden J.A., Mortensen X.M., Reddy A., Zhang A.Y. (2022). Systemic and Ocular Adverse Events with Intravitreal Anti-VEGF Therapy Used in the Treatment of Diabetic Retinopathy: A Review. Curr. Diabetes Rep..

[67] Adcock I.M., Mumby S. (2017). Glucocorticoids. Handb. Exp. Pharmacol..

[68] Kapugi M., Cunningham K. (2019). Corticosteroids. Orthop. Nurs..

[69] Jonas J.B., Söfker A. (2001). Intraocular injection of crystalline cortisone as adjunctive treatment of diabetic macular edema. Am. J. Ophthalmol..

[70] Jusufbegovic D., Schaal S. (2017). Quiescent herpes simplex keratitis reactivation after intravitreal injection of dexamethasone implant. Retin. Cases Brief Rep..

[71] Nicholson B.P., Atchison E., Idris A.A., Bakri S.J. (2018). Central serous chorioretinopathy and glucocorticoids: An update on evidence for association. Surv. Ophthalmol..

[72] Araki T., Ishikawa H., Iwahashi C., Niki M., Mitamura Y., Sugimoto M., Kondo M., Kinoshita T., Nishi T., Ueda T. (2019). Central serous chorioretinopathy with and without steroids: A multicenter survey. PLoS ONE.

[73] Kačmar J., Cholevík D. (2019). Corticosteroid Induced Posterior Subcapsular Cataract. Czech Slovak Ophthalmol..

[74] Roberti G., Oddone F., Agnifili L., Katsanos A., Michelessi M., Mastropasqua L., Quaranta L., Riva I., Tanga L., Manni G. (2020). Steroid-induced glaucoma: Epidemiology, pathophysiology, and clinical management. Surv. Ophthalmol..

[75] Ulavíková Z., Anwarzai J., Krásnik V. (2022). Acute retinal necrosis after intravitreal dexamethasone implant. A case report. Cesk. Slov. Oftalmol..

[76] Armaly M.F., Becker B. (1965). Intraocular pressure response to topical corticosteroids. Fed. Proc..

[77] Sohn H.J., Han D.H., Kim I.T., Oh I.K., Kim K.H., Lee D.Y., Nam D.H. (2011). Changes in aqueous concentrations of various cytokines after intravitreal triamcinolone versus bevacizumab for diabetic macular edema. Am. J. Ophthalmol..

[78] Hauser D., Bukelman A., Pokroy R., Katz H., Len A., Thein R., Parness-Yossifon R., Pollack A. (2008). Intravitreal triamcinolone for diabetic macular edema: Comparison of 1, 2, and 4 mg. Retina.

[79] Yilmaz T., Weaver C.D., Gallagher M.J., Cordero-Coma M., Cervantes-Castaneda R.A., Klisovic D., Lavaque A.J., Larson R.J. (2009). Intravitreal triamcinolone acetonide injection for treatment of refractory diabetic macular edema: A systematic review. Ophthalmology.

[80] Ogura Y., Shimura M., Iida T., Sakamoto T., Yoshimura N., Yamada M., Ishibashi T. (2019). Phase II/III Clinical Trial of Sub-Tenon Injection of Triamcinolone Acetonide (WP-0508ST) for Diabetic Macular Edema. Ophthalmologica.

[81] Nawar A.E. (2022). Effectiveness of Suprachoroidal Injection of Triamcinolone Acetonide in Resistant Diabetic Macular Edema Using a Modified Microneedle. Clin. Ophthalmol..

[82] Chang-Lin J.-E., Attar M., Acheampong A.A., Robinson M.R., Whitcup S.M., Kuppermann B.D., Welty D. (2011). Pharmacokinetics and pharmacodynamics of a sustained-release dexamethasone intravitreal implant. Investig. Ophthalmol. Vis. Sci..

[83] Campochiaro P.A., Nguyen Q.D., Hafiz G., Bloom S., Brown D.M., Busquets M., Ciulla T., Feiner L., Sabates N., Billman K. (2013). Aqueous levels of fluocinolone acetonide after administration of fluocinolone acetonide inserts or fluocinolone acetonide implants. Ophthalmology.

[84] Fusi-Rubiano W., Blow R.R., Lane M., Morjaria R., Denniston A.K. (2018). Iluvien™ (Fluocinolone Acetonide 0.19 mg Intravitreal Implant) in the Treatment of Diabetic Macular Edema: A Review. Ophthalmol. Ther..

[85] Kodjikian L., Bandello F., de Smet M., Dot C., Zarranz-Ventura J., Loewenstein A., Sudhalkar A., Bilgic A., Cunha-Vaz J., Dirven W. (2022). Fluocinolone acetonide implant in diabetic macular edema: International experts’ panel consensus guidelines and treatment algorithm. Eur. J. Ophthalmol..

[86] Audren F., Tod M., Massin P., Benosman R., Haouchine B., Erginay A., Caulin C., Gaudric A., Bergmann J.-F. (2004). Pharmacokinetic–pharmacodynamic modeling of the effect of triamcinolone acetonide on central macular thickness in patients with diabetic macular edema. Investig. Ophthalmol. Vis. Sci..

[87] Gillies M.C., Simpson J.M., Gaston C., Hunt G., Ali H., Zhu M., Sutter F. (2009). Five-year results of a randomized trial with open-label extension of triamcinolone acetonide for refractory diabetic macular edema. Ophthalmology.

[88] Aceves-Franco L.A., Sanchez-Aguilar O.E., Barragan-Arias A.R., Ponce-Gallegos M.A., Navarro-Partida J., Santos A. (2023). The Evolution of Triamcinolone Acetonide Therapeutic Use in Retinal Diseases: From Off-Label Intravitreal Injection to Advanced Nano-Drug Delivery Systems. Biomedicines.

[89] Zając-Pytrus H.M., Kaczmarek R., Strońska-Lipowicz D., Pomorska M., Misiuk-Hojło M. (2017). Theeffects and safety of intravitreal triamcinolone injections in the treatment of diabetic macular edema. Adv. Clin. Exp. Med..

[90] Sorrentino F.S., Bonifazzi C., Parmeggiani F. (2021). Diabetic macular edema: Safe and effective treatment with intravitreal triamcinolone acetonide (Taioftal). PLoS ONE.

[91] Rodrigues M.W., Cardillo J.A., Messias A., Siqueira R.C., Scott I.U., Jorge R. (2020). Bevacizumab versus triamcinolone for persistent diabetic macular edema: A randomized clinical trial. Graefes Arch. Clin. Exp. Ophthalmol..

[92] Abdel-Maboud M., Menshawy E., Bahbah E.I., Outani O., Menshawy A. (2021). Intravitreal bevacizumab versus intravitreal triamcinolone for diabetic macular edema–Systematic review, meta-analysis and meta-regression. PLoS ONE.

[93] Zhu Y., Li J., Yu S., Mao B., Ying J. (2022). Clinical Comparative Study of Intravitreal Injection of Triamcinolone Acetonide and Aflibercept in the Treatment of Diabetic Retinopathy Cystoid Macular Edema. Emerg. Med. Int..

[94] Jain S., Thompson J.R., Foot B., Tatham A., Eke T. (2014). Severe intraocular pressure rise following intravitreal triamcinolone: A national survey to estimate incidence and describe case profiles. Eye.

[95] Kriechbaum K., Prager S., Mylonas G., Scholda C., Rainer G., Funk M., Kundi M., Schmidt-Erfurth U., Diabetic Retinopathy Research Group (2014). Intravitreal bevacizumab (Avastin) versus triamcinolone (Volon A) for treatment of diabetic macular edema: One-year results. Eye.

[96] Soheilian M., Garfami K.H., Ramezani A., Yaseri M., Peyman G.A. (2012). Two-year results of a randomized trial of intravitreal bevacizumab alone or combined with triamcinolone versus laser in diabetic macular edema. Retina.

[97] Zhang L., Chen X. (2021). Efficacy and safety of triamcinolone acetonide injection combined with laser photocoagulation in the treatment of diabetic macular edema: A systematic review and meta-analysis. Ann. Palliat. Med..

[98] Arain M.A., Muzaffar W., Farooq O., Azhar M.N. (2018). Combined Intravitreal Triamcenolone Acetonide and Bevacizumab for Refractory Diabetic Macular Edema. J. Coll. Physicians Surg. Pak..

[99] Barakat M.R., Wykoff C.C., Gonzalez V., Hu A., Marcus D., Zavaleta E., Ciulla T.A. (2021). Suprachoroidal CLS-TA plus Intravitreal Aflibercept for Diabetic Macular Edema: A Randomized, Double-Masked, Parallel-Design, Controlled Study. Ophthalmol. Retin..

[100] Callanan D.G., Gupta S., Boyer D.S., Ciulla T.A., Singer M.A., Kuppermann B.D., Liu C.-C., Li X.-Y., Hollander D.A., Schiffman R.M. (2013). Dexamethasone intravitreal implant in combination with laser photocoagulation for the treatment of diffuse diabetic macular edema. Ophthalmology.

[101] Lam W.-C., Albiani D., Yoganathan P., Chen J.C., Kherani A., Maberley D., Oliver A., Rabinovitch T., Sheidow T.G., Tourville E. (2015). Real-world assessment of intravitreal dexamethasone implant (0.7 mg) in patients with macular edema: The CHROME study. Clin. Ophthalmol..

[102] Heng L.Z., Sivaprasad S., Crosby-Nwaobi R., Saihan Z., Karampelas M., Bunce C., Peto T., Hykin P.G. (2016). A prospective randomised controlled clinical trial comparing a combination of repeated intravitreal Ozurdex and macular laser therapy versus macular laser only in centre-involving diabetic macular oedema (OZLASE study). Br. J. Ophthalmol..

[103] Malclès A., Dot C., Voirin N., Agard E., Vié A.-L., Bellocq D., Denis P., Kodjikian L. (2017). Real-life study in diabetic macular edema treated with dexamethasone implant: The reldex study. Retina.

[104] Rosenblatt A., Udaondo P., Cunha-Vaz J., Sivaprasad S., Bandello F., Lanzetta P., Kodjikian L., Goldstein M., Habot-Wilner Z., Loewenstein A. (2020). A Collaborative Retrospective Study on the Efficacy and Safety of Intravitreal Dexamethasone Implant (Ozurdex) in Patients with Diabetic Macular Edema: The European DME Registry Study. Ophthalmology.

[105] Kodjikian L., Bellocq D., Mathis T. (2018). Pharmacological Management of Diabetic Macular Edema in Real-Life Observational Studies. BioMed Res. Int..

[106] Mehta H., Nguyen V., Barthelmes D., Pershing S., Chi G.C., Dopart P., Gillies M.C. (2022). Outcomes of Over 40,000 Eyes Treated for Diabetic Macula Edema in Routine Clinical Practice: A Systematic Review and Meta-analysis. Adv. Ther..

[107] Callanan D.G., Loewenstein A., Patel S.S., Massin P., Corcóstegui B., Li X.-Y., Jiao J., Hashad Y., Whitcup S.M. (2017). A multicenter, 12-month randomized study comparing dexamethasone intravitreal implant with ranibizumab in patients with diabetic macular edema. Graefes Arch. Clin. Exp. Ophthalmol..

[108] Cornish E.E., Teo K.Y., Gillies M.C., Lim L.L., Nguyen V., Wickremasinghe S., Mehta H., McAllister I.L., Fraser-Bell S. (2022). Five-year outcomes of eyes initially enrolled in the 2-year BEVORDEX trial of bevacizumab or dexamethasone implants for diabetic macular oedema. Br. J. Ophthalmol..

[109] Al-Khersan H., Hariprasad S.M., Chhablani J. (2017). Dex Implant Study Group. Early Response to Intravitreal Dexamethasone Implant Therapy in Diabetic Macular Edema May Predict Visual Outcome. Am. J. Ophthalmol..

[110] Bucolo C., Gozzo L., Longo L., Mansueto S., Vitale D.C., Drago F. (2018). Long-term efficacy and safety profile of multiple injections of intravitreal dexamethasone implant to manage diabetic macular edema: A systematic review of real-world studies. J. Pharmacol. Sci..

[111] Castro-Navarro V., Cervera-Taulet E., Navarro-Palop C., Monferrer-Adsuara C., Hernández-Bel L., Montero-Hernández J. (2019). Intravitreal dexamethasone implant Ozurdex^®^ in naïve and refractory patients with different subtypes of diabetic macular edema. BMC Ophthalmol..

[112] Zarranz-Ventura J., Romero-Núñez B., Bernal-Morales C., Velazquez-Villoria D., Sala-Puigdollers A., Figueras-Roca M., Copete S., Distefano L., Boixadera A., García-Arumi J. (2020). Differential response to intravitreal dexamethasone implant in naïve and previously treated diabetic macular edema eyes. BMC Ophthalmol..

[113] Sarao V., Veritti D., Furino C., Giancipoli E., Alessio G., Boscia F., Lanzetta P. (2017). Dexamethasone implant with fixed or individualized regimen in the treatment of diabetic macular oedema: Six-month outcomes of the UDBASA study. Acta Ophthalmol..

[114] Malclès A., Dot C., Voirin N., Vié A.-L., Agard E., Bellocq D., Denis P., Kodjikian L. (2017). Safety of intravitreal dexamethasone implant (ozurdex): The SAFODEX study. Incidence and Risk Factors of Ocular Hypertension. Retina.

[115] Maturi R.K., Glassman A.R., Liu D., Beck R.W., Bhavsar A.R., Bressler N.M., Jampol L.M., Melia M., Punjabi O.S., Salehi-Had H. (2018). Effect of Adding Dexamethasone to Continued Ranibizumab Treatment in Patients With Persistent Diabetic Macular Edema: A DRCR Network Phase 2 Randomized Clinical Trial. JAMA Ophthalmol..

[116] Martínez A.H., Delgado E.P., Silva G.S., Mateos L.C., Pascual J.L., Villa J.L., Vicente P.G., Almeida-González C.-V. (2020). Early versus late switch: How long should we extend the anti-vascular endothelial growth factor therapy in unresponsive diabetic macular edema patients?. Eur. J. Ophthalmol..

[117] Rajesh B., Zarranz-Ventura J., Fung A.T., Busch C., Sahoo N.K., Rodriguez-Valdes P.J., Sarao V., Mishra S.K., Saatci A.O., Mirete P.U. (2020). Safety of 6000 intravitreal dexamethasone implants. Br. J. Ophthalmol..

[118] Pérez-Sarriegui A., Casas-Llera P., Díez-Álvarez L., Contreras I., Moreno-López M., Figueroa M., González-Martín-Moro J., Muñoz-Negrete F., Rebolleda G. (2018). Phaco-non-penetrating deep sclerectomy in ocular hypertension secondary to dexamethasone intravitreal implant. Arch. Soc. Esp. Oftalmol..

[119] Kaya M., Atas F., Kocak N., Ozturk T., Ayhan Z., Kaynak S. (2023). Intravitreal Ranibizumab and Dexamethasone Implant Injections as Primary Treatment of Diabetic Macular Edema: The Month 24 Results from Simultaneously Double Protocol. Curr. Eye Res..

[120] He Y., Ren X.-J., Hu B.-J., Lam W.-C., Li X.-R. (2018). A meta-analysis of the effect of a dexamethasone intravitreal implant versus intravitreal anti-vascular endothelial growth factor treatment for diabetic macular edema. BMC Ophthalmol..

[121] Shah S.U., Harless A., Bleau L., Maturi R.K. (2016). Prospective randomized subject-masked study of intravitreal bevacizumab monotherapy versus dexamethasone implant monotherapy in the treatment of persistent diabetic macular edema. Retina.

[122] Comet A., Gascon P., Ramtohul P., Donnadieu B., Denis D., Matonti F. (2021). INVICTUS: Intravitreal anti-VEGF and dexamethasone implant comparison for the treatment of diabetic macular edema: A 12 months follow-up study. Eur. J. Ophthalmol..

[123] Patil N.S., Mihalache A., Hatamnejad A., Popovic M.M., Kertes P.J., Muni R.H. (2023). Intravitreal Steroids Compared with Anti-VEGF Treatment for Diabetic Macular Edema: A Meta-Analysis. Ophthalmol. Retin..

[124] Chi S.-C., Kang Y.-N., Huang Y.-M. (2023). Efficacy and safety profile of intravitreal dexamethasone implant versus antivascular endothelial growth factor treatment in diabetic macular edema: A systematic review and meta-analysis. Sci. Rep..

[125] Pacella F., Romano M.R., Turchetti P., Tarquini G., Carnovale A., Mollicone A., Mastromatteo A., Pacella E. (2016). An eighteen-month follow-up study on the effects of Intravitreal Dexamethasone Implant in diabetic macular edema refractory to anti-VEGF therapy. Int. J. Ophthalmol..

[126] Veritti D., Sarao V., Soppelsa V., Lanzetta P. (2021). Managing Diabetic Macular Edema in Clinical Practice: Systematic Review and Meta-Analysis of Current Strategies and Treatment Options. Clin. Ophthalmol..

[127] Holekamp N.M., Campbell J., Almony A., Ingraham H., Marks S., Chandwani H., Cole A.L., Kiss S. (2018). Vision Outcomes Following Anti–Vascular Endothelial Growth Factor Treatment of Diabetic Macular Edema in Clinical Practice. Am. J. Ophthalmol..

[128] Ciulla T.A., Pollack J.S., Williams D.F. (2021). Visual acuity outcomes and anti-VEGF therapy intensity in diabetic macular oedema: A real-world analysis of 28 658 patient eyes. Br. J. Ophthalmol..

[129] Mitchell P., Bandello F., Schmidt-Erfurth U., Lang G.E., Massin P., Schlingemann R.O., Sutter F., Simader C., Burian G., Gerstner O. (2011). The RESTORE study: Ranibizumab monotherapy or combined with laser versus laser monotherapy for diabetic macular edema. Ophthalmology.

[130] Gascon P., Borget I., Comet A., Carton L., Matonti F., Dupont-Benjamin L. (2021). Costs comparison of treating diabetic macular edema with aflibercept, ranibizumab or dexamethasone at 1 year in France (INVICOST study). Eur. J. Ophthalmol..

[131] Busch C., Zur D., Fraser-Bell S., Laíns I., Santos A.R., Lupidi M., Cagini C., Gabrielle P.-H., Couturier A., Mané-Tauty V. (2018). Shall we stay, or shall we switch? Continued anti-VEGF therapy versus early switch to dexamethasone implant in refractory diabetic macular edema. Acta Diabetol..

[132] Ruiz-Moreno J.M., Ruiz-Medrano J. (2023). Early-switch versus late-switch in patients with diabetic macular edema: A cost-effectiveness study. Graefes Arch. Clin. Exp. Ophthalmol..

[133] Peto T. (2017). An overview of the clinical outcomes of the fluocinolone acetonide 190 µg intravitreal implant clinical evidence study in the United Kingdom (ICE-UK). Curr. Med. Res. Opin..

[134] Eaton A., Koh S.S., Jimenez J., Riemann C.D. (2019). The USER Study: A Chart Review of Patients Receiving a 0.2 µg/day Fluocinolone Acetonide Implant for Diabetic Macular Edema. Ophthalmol. Ther..

[135] Augustin A.J., Bopp S., Fechner M., Holz F., Sandner D., Winkgen A.-M., Khoramnia R., Neuhann T., Warscher M., Spitzer M. (2020). Three-year results from the Retro-IDEAL study: Real-world data from diabetic macular edema (DME) patients treated with ILUVIEN^®^ (0.19 mg fluocinolone acetonide implant). Eur. J. Ophthalmol..

[136] Rehak M., Busch C., Unterlauft J.-D., Jochmann C., Wiedemann P. (2020). Outcomes in diabetic macular edema switched directly or after a dexamethasone implant to a fluocinolone acetonide intravitreal implant following anti-VEGF treatment. Acta Diabetol..

[137] Cicinelli M.V., Rosenblatt A., Grosso D., Zollet P., Capone L., Rabiolo A., Lattanzio R., Loewenstein A., Bandello F., Nassisi M. (2021). The outcome of fluocinolone acetonide intravitreal implant is predicted by the response to dexamethasone implant in diabetic macular oedema. Eye.

[138] Bailey C., Chakravarthy U., Lotery A., Menon G., Talks J. (2022). Medisoft Audit Group Extended real-world experience with the ILUVIEN^®^ (fluocinolone acetonide) implant in the United Kingdom: 3-year results from the Medisoft^®^ audit study. Eye.

[139] Baillif S., Staccini P., Weber M., Delyfer M.-N., Le Mer Y., Gualino V., Collot L., Merite P.-Y., Creuzot-Garcher C., Kodjikian L. (2022). Management of Patients with Diabetic Macular Edema Switched from Dexamethasone Intravitreal Implant to Fluocinolone Acetonide Intravitreal Implant. Pharmaceutics.

[140] Mathis T., Papegaey M., Ricard C., Rezkallah A., Matonti F., Sudhalkar A., Vartin C., Dot C., Kodjikian L. (2022). Efficacy and Safety of Intravitreal Fluocinolone Acetonide Implant for Chronic Diabetic Macular Edema Previously Treated in Real-Life Practice: The REALFAc Study. Pharmaceutics.

[141] Singer M.A., Sheth V., Mansour S.E., Coughlin B., Gonzalez V.H. (2022). Three-Year Safety and Efficacy of the 0.19-mg Fluocinolone Acetonide Intravitreal Implant for Diabetic Macular Edema: The PALADIN Study. Ophthalmology.

[142] Fallico M., Maugeri A., Lotery A., Longo A., Bonfiglio V., Russo A., Avitabile T., Furino C., Cennamo G., Barchitta M. (2021). Fluocinolone acetonide vitreous insert for chronic diabetic macular oedema: A systematic review with meta-analysis of real-world experience. Sci. Rep..

[143] Kodjikian L., Baillif S., Creuzot-Garcher C., Delyfer M.-N., Matonti F., Weber M., Mathis T. (2021). Real-World Efficacy and Safety of Fluocinolone Acetonide Implant for Diabetic Macular Edema: A Systematic Review. Pharmaceutics.

[144] Khoramnia R., Peto T., Koch F., Taylor S.R., de Sousa J.P.C., Hill L., Bailey C., Chakravarthy U., ILUVIEN Registry Safety Study (IRISS) Investigators Group (2023). Safety and effectiveness of the fluocinolone acetonide intravitreal implant (ILUVIEN): 3-year results from the European IRISS registry study. Br. J. Ophthalmol..

[145] Schmit-Eilenberger V. (2015). A novel intravitreal fluocinolone acetonide implant (Iluvien^®^) in the treatment of patients with chronic diabetic macular edema that is insufficiently responsive to other medical treatment options: A case series. Clin. Ophthalmol..

[146] McCluskey J.D., Kaufman P.L., Wynne K., Lewis G. (2019). Early adoption of the fluocinolone acetonide (FAc) intravitreal implant in patients with persistent or recurrent diabetic macular edema (DME). Int. Med. Case Rep. J..

[147] Ch’ng S.W., Brent A.J., Empeslidis T., Konidaris V., Banerjee S. (2018). Real-World Cost Savings Demonstrated by Switching Patients with Refractory Diabetic Macular Edema to Intravitreal Fluocinolone Acetonide (Iluvien): A Retrospective Cost Analysis Study. Ophthalmol. Ther..

[148] Parrish R.K., Campochiaro P.A., Pearson P.A., Green K., Traverso C.E. (2016). FAME Study Group Characterization of Intraocular Pressure Increases and Management Strategies Following Treatment With Fluocinolone Acetonide Intravitreal Implants in the FAME Trials. Ophthalmic Surg. Lasers Imaging Retin..

[149] Roth D.B., Eichenbaum D., Malik D., Radcliffe N.M., Cutino A., Small K.W., PALADIN Study Group (2023). The 0.19-mg Fluocinolone Acetonide Intravitreal Implant for Diabetic Macular Edema: Intraocular Pressure-Related Effects over 36 Months. Ophthalmol. Retin..

[150] El Rayes E.N., Leila M. (2023). Visual function and retinal morphological changes after single suprachoroidal delivery of fluocinolone acetonide (Iluvien^®^) implant in eyes with chronic diabetic macular edema. Int. J. Retin. Vitr..

[151] Bressler S.B., Odia I., Maguire M.G., Dhoot D.S., Glassman A.R., Jampol L.M., Marcus D.M., Solomon S.D., Sun J.K., Diabetic Retinopathy Clinical Research Network (2022). Factors Associated With Visual Acuity and Central Subfield Thickness Changes When Treating Diabetic Macular Edema With Anti–Vascular Endothelial Growth Factor Therapy: An Exploratory Analysis of the Protocol T Randomized Clinical Trial. JAMA Ophthalmol..

[152] Kwon J.-W., Jee D. (2018). Aqueous humor cytokine levels in patients with diabetic macular edema refractory to anti-VEGF treatment. PLoS ONE.

[153] Ehlken C., Helms M., Böhringer D., Agostini H.T., Stahl A. (2017). Association of treatment adherence with real-life VA outcomes in AMD, DME, and BRVO patients. Clin. Ophthalmol..

[154] Uludag G., Hassan M., Matsumiya W., Pham B.H., Chea S., Trong Tuong Than N., Doan H.L., Akhavanrezayat A., Halim M.S., Do D.V. (2022). Efficacy and safety of intravitreal anti-VEGF therapy in diabetic retinopathy: What we have learned and what should we learn further?. Expert Opin. Biol. Ther..

[155] Polizzi S., Mahajan V.B. (2015). Intravitreal Anti-VEGF Injections in Pregnancy: Case Series and Review of Literature. J. Ocul. Pharmacol. Ther..

[156] Fazelat A., Lashkari K. (2011). Off-label use of intravitreal triamcinolone acetonide for diabetic macular edema in a pregnant patient. Clin. Ophthalmol..

[157] Concillado M., Lund-Andersen H., Mathiesen E.R., Larsen M. (2016). Dexamethasone Intravitreal Implant for Diabetic Macular Edema During Pregnancy. Am. J. Ophthalmol..

[158] Sophie R., Lu N., Campochiaro P.A. (2015). Predictors of Functional and Anatomic Outcomes in Patients with Diabetic Macular Edema Treated with Ranibizumab. Ophthalmology.

[159] Terada N., Murakami T., Uji A., Dodo Y., Mori Y., Tsujikawa A. (2020). Hyperreflective Walls in Foveal Cystoid Spaces as a Biomarker of Diabetic Macular Edema Refractory to Anti-VEGF Treatment. Sci. Rep..

[160] Vujosevic S., Simó R. (2017). Local and Systemic Inflammatory Biomarkers of Diabetic Retinopathy: An Integrative Approach. Investig. Ophthalmol. Vis. Sci..

[161] Jeng C.-J., Hsieh Y.-T., Yang C.-M., Yang C.-H., Lin C.-L., Wang I.-J. (2018). Development of diabetic retinopathy after cataract surgery. PLoS ONE.

[162] Khodabandeh A., Fadaifard S., Abdollahi A., Karkhaneh R., Roohipoor R., Abdi F., Ghasemi H., Habibollahi S., Mazloumi M. (2018). Role of combined phacoemulsification and intravitreal injection of bevacizumab in prevention of postoperative macular edema in non-proliferative diabetic retinopathy. J. Curr. Ophthalmol..

[163] Tatsumi T., Oshitari T., Ando T., Takatsuna Y., Arai M., Baba T., Sato E., Yamamoto S. (2018). Comparison of the Efficacy of Sub-Tenon versus Intravitreal Triamcinolone Acetonide Injection during Cataract Surgery for Diabetic Macular Edema. Ophthalmologica.

[164] Fallico M., Avitabile T., Castellino N., Longo A., Russo A., Bonfiglio V., Parisi F., Furino C., Panozzo G., Scorcia V. (2021). Intravitreal dexamethasone implant one month before versus concomitant with cataract surgery in patients with diabetic macular oedema: The dexcat study. Acta Ophthalmol..

[165] Furino C., Boscia F., Niro A., D’Addario M., Grassi M.O., Saglimbene V., Reibaldi M., Alessio G. (2021). Diabetic macular edema and cataract surgery: Phacoemulsification Combined With Dexamethasone Intravitreal Implant Compared With Standard Phacoemulsification. Retina.

[166] Lee S.S., Ghosn C., Yu Z., Zacharias L.C., Kao H., Lanni C., Abdelfattah N., Kuppermann B., Csaky K.G., D’Argenio D.Z. (2010). Vitreous VEGF Clearance is increased after vitrectomy. Investig. Ophthalmol. Vis. Sci..

[167] Costa J.F., Sousa K., Marques J.P., Marques M., Cachulo M.L., Silva R., Gomes N., Figueira J. (2016). Efficacy and safety of postvitrectomy intravitreal triamcinolone therapy for diabetic macular edema. Eur. J. Ophthalmol..

[168] Watanabe A., Tsuzuki A., Arai K., Gekka T., Kohzaki K., Tsuneoka H. (2016). Efficacy of Intravitreal Triamcinolone Acetonide for Diabetic Macular Edema After Vitrectomy. J. Ocul. Pharmacol. Ther..

[169] Rezkallah A., Malclès A., Dot C., Voirin N., Agard É., Vié A.-L., Denis P., Mathis T., Kodjikian L. (2017). Evaluation of efficacy and safety of dexamethasone intravitreal implants before and after vitrectomy in a real-life study. Acta Ophthalmol..

[170] Pessoa B., Coelho J., Correia N., Ferreira N., Beirão M., Meireles A. (2017). Fluocinolone Acetonide Intravitreal Implant 190 μg (ILUVIEN^®^) in Vitrectomized versus Nonvitrectomized Eyes for the Treatment of Chronic Diabetic Macular Edema. Ophthalmic Res..

[171] Breusegem C., Vandewalle E., Van Calster J., Stalmans I., Zeyen T. (2009). Predictive value of a topical dexamethasone provocative test before intravitreal triamcinolone acetonide injection. Investig. Ophthalmol. Vis. Sci..

[172] Tsoutsanis P., Kapantais D. (2023). Anterior migration of Ozurdex implant: A review on risk factors, complications, and management. Int. J. Retin. Vitr..

[173] Rishi P., Majumder P.D., Biswas J. (2019). Anterior Chamber Migration of Fluocinolone Acetonide Intravitreal Implant. JAMA Ophthalmol..

[174] Wykoff C.C., Chakravarthy U., Campochiaro P.A., Bailey C., Green K., Cunha-Vaz J. (2017). Long-term Effects of Intravitreal 0.19 mg Fluocinolone Acetonide Implant on Progression and Regression of Diabetic Retinopathy. Ophthalmology.

[175] Iglicki M., Zur D., Busch C., Okada M., Loewenstein A. (2018). Progression of diabetic retinopathy severity after treatment with dexamethasone implant: A 24-month cohort study the ‘DR-Pro-DEX Study’. Acta Diabetol..

[176] Rittiphairoj T., Mir T.A., Li T., Virgili G. (2020). Intravitreal steroids for macular edema in diabetes. Cochrane Database Syst. Rev..

[177] Zarranz-Ventura J., Mali J.O. (2020). Effectiveness of 190 μg Fluocinolone Acetonide and 700 μg Dexamethasone Intravitreal Implants in Diabetic Macular Edema Using the Area-Under-the-Curve Method: The CONSTANT Analysis. Clin. Ophthalmol..

[178] Kuley B., Storey P.P., Pancholy M., Wibbelsman T.D., Obeid A., Regillo C., Garg S. (2020). Treatment of Eyes With Diabetic Macular Edema That Had a Suboptimal Response to Antivascular Endothelial Growth Factor Therapy: 2-mg Intravitreal Triamcinolone Acetonide vs. 0.7-mg Dexamethasone Implant. J. Vitr. Dis..

[179] Augustin A.J., Kuppermann B.D., Lanzetta P., Loewenstein A., Li X.-Y., Cui H., Hashad Y., Whitcup S.M., Ozurdex MEAD Study Group (2015). Dexamethasone intravitreal implant in previously treated patients with diabetic macular edema: Subgroup analysis of the MEAD study. BMC Ophthalmol..

[180] Thakur A., Kadam R., Kompella U.B. (2011). Trabecular Meshwork and Lens Partitioning of Corticosteroids: Implications for elevated intraocular pressure and cataracts. Arch. Ophthalmol..

[181] Amoaku W.M.K., Saker S., Stewart E.A. (2015). A review of therapies for diabetic macular oedema and rationale for combination therapy. Eye.

[182] Ehlers J.P., Yeh S., Maguire M.G., Smith J.R., Mruthyunjaya P., Jain N., Kim L.A., Weng C.Y., Flaxel C.J., Schoenberger S.D. (2021). Intravitreal Pharmacotherapies for Diabetic Macular Edema: A Report by the American Academy of Ophthalmology. Ophthalmology.

[183] Mehta H., Hennings C., Gillies M.C., Nguyen V., Campain A., Fraser-Bell S. (2018). Anti-vascular endothelial growth factor combined with intravitreal steroids for diabetic macular oedema. Cochrane Database Syst. Rev..

[184] Weinberg T., Loewenstein A. (2020). The role of steroids in treating diabetic macular oedema in the era of anti-VEGF. Eye.

[185] Mansour S.E., Browning D.J., Wong K., Flynn H.W., Bhavsar A.R. (2020). The Evolving Treatment of Diabetic Retinopathy. Clin. Ophthalmol..

